# Identification of Potential Biomarkers Associated with Impaired Fatty Acid Oxidation in Aged Skeletal Muscle Using Bioinformatics and Machine Learning Approaches

**DOI:** 10.3390/biom16071030

**Published:** 2026-07-14

**Authors:** Haoyang Gao, Fangjie Yang, Jiabin Wu, Minghao Ji, Xiaotong Ma, Danlin Zhu, Linlin Zhao, Weihua Xiao

**Affiliations:** 1 Shanghai Key Lab of Human Performance, Shanghai University of Sport, 650 Qingyuan Ring Road, Yangpu District, Shanghai 200438, China; haoyanggao1999@163.com (H.G.); 2511517001@sus.edu.cn (F.Y.); 2511517006@sus.edu.cn (J.W.); 2421516010@sus.edu.cn (M.J.); 2321518007@sus.edu.cn (X.M.); 2 School of Humanities and Social Sciences, Shanghai Lida University, Shanghai 201609, China; zdl981030@163.com; 3 School of Physical Education, Shanghai Normal University, Shanghai 200234, China

**Keywords:** aging, sarcopenia, fatty acid oxidation, biomarker, machine learning, artificial neural network

## Abstract

**Objective**: Impaired fatty acid oxidation (FAO) is considered an important metabolic mechanism underlying skeletal muscle aging and sarcopenia; however, the key regulatory molecules involved in this process remain incompletely defined. This study aimed to identify candidate biomarkers associated with impaired FAO in aged skeletal muscle, characterize their potential biological functions and regulatory features through integrated bioinformatics and machine learning analyses, and preliminarily validate their expression patterns in in vivo and in vitro aging models. **Methods**: Skeletal muscle aging transcriptomic datasets GSE1428 and GSE674 were obtained from the Gene Expression Omnibus database. FAO-related genes were retrieved from GeneCards. Differentially expressed FAO-related genes (DE-FAOGs) were identified through differential expression analysis and were further analyzed by Gene Ontology and Kyoto Encyclopedia of Genes and Genomes enrichment analyses. Random forest, Boruta, and protein–protein interaction (PPI) network analyses were used to screen hub genes, and an artificial neural network (ANN) model was constructed. Single-cell RNA sequencing analysis, gene set enrichment analysis, ceRNA network construction, drug prediction, molecular docking, and molecular dynamics simulation were further performed. Hub gene expression was validated by qRT-PCR in naturally aged mice and D-galactose-induced senescent C2C12 cells. **Results**: A total of 69 DE-FAOGs were identified and were mainly enriched in mitochondrial function, electron transport chain, and energy metabolism-related pathways. Three hub genes, creatine kinase, mitochondrial 2 (*CKMT2*), actin alpha cardiac muscle 1 (*ACTC1*), and forkhead box O3 (*FOXO3*), were identified by random forest, Boruta, and PPI analyses. Receiver operating characteristic (ROC) analysis showed good discriminatory performance for these genes. The three-gene ANN model achieved area under the curve (AUC) values of 0.992 and 0.964 in the training and validation datasets, respectively. Gene set enrichment analysis (GSEA) suggested that the hub genes were closely associated with mitochondrial energy metabolism, lipid metabolism, and stress regulation. qRT-PCR confirmed decreased Ckmt2 expression and increased Actc1 and Foxo3 expression under aging conditions, consistent with the bioinformatics results. **Conclusions**: *CKMT2*, *ACTC1*, and *FOXO3* are potential biomarkers associated with impaired FAO in aged skeletal muscle. The ANN model based on these three genes showed good predictive performance and may provide new insights into the metabolic mechanisms and therapeutic targets of sarcopenia.

## 1. Introduction

As the proportion of older adults continues to increase, population aging has become a long-term structural challenge for global health systems and chronic disease prevention. According to recent projections, global life expectancy is expected to continue increasing from 2022 to 2050, accompanied by a marked acceleration of population aging [1]. China entered an aging society in 2000. Statistical data showed that, in 2020, approximately 250 million people in China were aged 60 years or older, accounting for 17.8% of the total population [2]. By 2050, the proportion of individuals aged 80 years or older in China is projected to reach 10.3%, making China one of the countries with the highest proportion of older adults worldwide [2]. As a key organ responsible for maintaining locomotor capacity, metabolic homeostasis, and physiological reserve, skeletal muscle is highly vulnerable to aging and undergoes progressive declines in muscle mass, strength, and physical performance with advancing age [3]. Sarcopenia represents the clinical manifestation of this process. Its conservative prevalence is approximately 5–10% in the general population and is higher among older adults; it is closely associated with increased risks of falls, fractures, disability, hospitalization, and premature mortality [3]. Globally, sarcopenia has been reported to affect 10–16% of individuals older than 60 years [4], and its prevalence exceeds 50% among those older than 80 years [5]. The metabolic and functional impairments, as well as the disability risk caused by sarcopenia, have become major health concerns for older adults and have contributed to the increasing global disease burden. However, current clinical interventions for sarcopenia still largely rely on resistance exercise, nutritional support, and comprehensive lifestyle management, and there remains no widely accepted and approved pharmacological therapy that effectively improves the core functional outcomes of sarcopenia [6]. Although some hormone-related candidate therapies can increase lean body mass, their effects on clinically meaningful endpoints such as muscle strength, gait speed, and physical performance remain inconsistent, limiting their translational application [6]. Therefore, elucidating skeletal muscle molecular alterations in the context of aging is of both fundamental biological importance and clear public health and translational relevance.

Skeletal muscle is a major site of whole-body energy expenditure and substrate turnover. In particular, during resting, fasting, and endurance exercise conditions, mitochondrial fatty acid oxidation (FAO) is essential for ATP production, lipid homeostasis, and the maintenance of insulin sensitivity [7]. This process depends on the coordinated regulation of long-chain fatty acid transmembrane transport and mitochondrial uptake, carnitine palmitoyltransferase 1 (CPT1)-mediated carnitine shuttle activity, and energy-sensing and transcriptional regulatory networks, including adenosine monophosphate-activated protein kinase (AMPK)–acetyl-CoA carboxylase (ACC) and peroxisome proliferator-activated receptor (PPAR)/peroxisome proliferator-activated receptor gamma coactivator 1-alpha (PGC-1α) signaling [8,9,10]. Notably, skeletal muscle FAO is regulated not only by endogenous lipid mobilization but also by the availability of dietary fatty acids. Dietary fatty acids can serve as important exogenous substrates for oxidative energy production in skeletal muscle and participate in mitochondrial β-oxidation and ATP generation during fasting, exercise, and increased energy demand [7]. However, when dietary lipid intake or circulating fatty acid supply exceeds the mitochondrial oxidative capacity of skeletal muscle, lipid intermediates, acylcarnitines, and other metabolites may accumulate within muscle, thereby promoting lipotoxicity, insulin resistance, and increased mitochondrial metabolic burden [11,12]. Therefore, maintaining a balance among dietary fatty acid supply, skeletal muscle FAO capacity, and mitochondrial metabolic adaptation is important for preserving muscle energy homeostasis during aging. Increasing evidence from human and animal studies has shown that aged skeletal muscle exhibits reduced mitochondrial respiratory capacity, impaired coupling between intramyocellular lipid droplets and mitochondria, remodeling of oxidative phosphorylation and fatty acid degradation programs, and decreased metabolic flexibility [13,14]. Our previous study also showed that aged mice displayed age-related reductions in skeletal muscle mass and function, accompanied by disrupted mitochondrial quality control homeostasis [15]. In addition, aging is often accompanied by increased visceral adiposity and enhanced fatty infiltration within skeletal muscle [11]. When lipid supply exceeds the oxidative capacity of skeletal muscle, incomplete β-oxidation may occur, leading to the accumulation of lipid intermediates, including lipids and acylcarnitines, thereby inducing lipotoxicity and further promoting muscle functional decline and metabolic abnormalities [11]. These findings indicate that impaired FAO is not only a metabolic feature of skeletal muscle aging but also an important pathological mechanism linking mitochondrial dysfunction, lipid imbalance, and sarcopenic phenotypes.

However, impaired FAO in aged skeletal muscle is characterized by substantial multifactorial complexity and heterogeneity, making it difficult for single-candidate-gene strategies to reliably identify biomarkers with translational potential. With the continuous expansion of public databases such as the Gene Expression Omnibus (GEO), as well as the rapid accumulation of transcriptomic and single-cell omics data, bioinformatics frameworks based on differential expression analysis, gene set enrichment analysis (GSEA), protein–protein interaction (PPI) networks, and multi-algorithm feature selection have become important approaches for decoding the molecular landscape of skeletal muscle aging [16,17,18]. Further integration of machine learning methods, including random forest, LASSO, support vector machine (SVM), and artificial neural network (ANN) models, enables the extraction of discriminative feature combinations from high-dimensional data, followed by validation using external datasets and experimental models, thereby improving the reliability and interpretability of candidate biomarkers [16,17,18]. Based on this rationale, the present study aimed to identify candidate biomarkers associated with impaired FAO in aged skeletal muscle by integrating public transcriptomic datasets, FAO-related gene sets, bioinformatics analyses, and machine learning algorithms. We further evaluated their biological functions, cell population-specific expression patterns, predicted ceRNA regulatory networks, drug-related interaction profiles, and dynamic protein–ligand stability, followed by preliminary validation in naturally aged mouse skeletal muscle and D-galactose-induced senescent C2C12 myotubes. This integrated strategy was intended to provide candidate molecular signatures and translational clues for understanding FAO-related metabolic dysfunction in skeletal muscle aging and sarcopenia.

## 2. Materials and Methods

### 2.1. Data Collection and Preprocessing

The Gene Expression Omnibus (GEO) database (https://www.ncbi.nlm.nih.gov/geo/ [accessed on 3 December 2025]) was systematically searched using the keywords “aging” and “sarcopenia”, with a focus on the categories “Series”, “Homo sapiens”, and “Expression profiling by array”. Probe IDs were converted into gene symbols using Perl scripts, and the raw data from the analytical datasets were subsequently normalized using R software (version 4.4.1). The GSE1428 dataset was used as the training dataset, whereas the GSE674 dataset was used as the validation dataset. Details of the datasets used in this study are summarized in Table 1. Both datasets were derived from transcriptomic expression profiles of the vastus lateralis muscle. Fatty acid oxidation-related genes (FAOGs) were retrieved from the GeneCards database (https://www.genecards.org/ [accessed on 3 December 2025]) using the keyword “fatty acid oxidation”, with the gene type restricted to “protein coding”. A total of 1783 FAOGs were identified and are listed in Supplementary Material S2.

### 2.2. Identification of Differentially Expressed FAOGs

The R package “limma” was used to identify differentially expressed genes (DEGs) in the vastus lateralis muscle. The screening criteria were set as |log2| fold change (log2FC)| ≥ 0.585 and *p* < 0.05. A volcano plot was generated to visualize the distribution of DEGs between the older and younger groups. To explore the pathological mechanisms of skeletal muscle aging and the potential role of fatty acid oxidation in this process, DEGs were intersected with FAOGs to identify differentially expressed FAOGs (DE-FAOGs). The R package “pheatmap” was used to generate a heatmap for visualizing the expression patterns of DE-FAOGs.

### 2.3. Gene Ontology and Kyoto Encyclopedia of Genes and Genomes Enrichment Analyses

Gene Ontology (GO) and Kyoto Encyclopedia of Genes and Genomes (KEGG) pathway enrichment analyses were performed in R using the “org.Hs.eg.db” and “clusterProfiler” packages. GO terms and KEGG pathways with *p* < 0.05 were considered statistically significant. The top 10 enriched GO terms in the categories of biological process (BP), molecular function (MF), and cellular component (CC), as well as the top 20 enriched KEGG pathways, were visualized using the online bioinformatics platform (https://www.bioinformatics.com.cn [accessed on 10 March 2026]).

### 2.4. Screening of Candidate Feature Genes and Consensus Hub Biomarkers

To identify key feature genes associated with impaired fatty acid oxidation in aged skeletal muscle, random forest, Boruta algorithm, and PPI network analyses were jointly applied. Random forest is an ensemble learning algorithm based on multiple decision trees and can be used to evaluate the contribution of each variable to a classification task. In this study, the R package “randomForest” was used to construct the random forest model, with the parameter ntree set to 500. The top 10 genes ranked by gene importance score were selected as candidate feature genes. Boruta is a wrapper feature selection algorithm based on random forest that identifies stable and discriminative features by comparing the importance of real variables with that of randomly generated shadow variables. The R package “Boruta” was used for feature selection and for generating the gene importance evolution plot, in which confirmed important features were marked in green. Meanwhile, DE-FAOGs were imported into the STRING database to construct a PPI network. The species was set to Homo sapiens, and the minimum interaction confidence score was set to 0.4. The PPI results in TSV format were then imported into Cytoscape 3.9.1. The cytoHubba plugin was used to calculate node connectivity based on the Degree algorithm, and the top 10 genes ranked by Degree value were selected as candidate core genes in the PPI network. Finally, a Venn diagram was used to intersect the candidate genes obtained from random forest, Boruta, and PPI network analyses, thereby identifying the final consensus hub genes (http://bioinfogp.cnb.csic.es/tools/venny/index.html [accessed on 16 March 2026]).

### 2.5. Validation of Hub Genes

Receiver operating characteristic (ROC) curve models for the hub genes were constructed in both the training and validation datasets using R. The area under the curve (AUC) was calculated to preliminarily evaluate the diagnostic potential of the hub genes for skeletal muscle aging. An AUC value of 0.5–0.7 was considered to indicate low accuracy, 0.7–0.9 moderate accuracy, and ≥0.9 high accuracy. In addition, boxplots were generated using the “ggpubr” package to determine the expression levels of the hub genes in the validation dataset. A value of *p* < 0.05 was considered to indicate a statistically significant difference in gene expression between the older and young groups.

### 2.6. Construction and Validation of the ANN Diagnostic Model

The expression levels of the hub genes were used as input data and normalized using the min–max method. The hub gene scores were then assigned according to the expression direction of each gene in the older group. For hub genes upregulated in the older group, samples with expression levels higher than the median were assigned a score of 1, whereas those with expression levels lower than or equal to the median were assigned a score of 0. Conversely, for hub genes downregulated in the older group, samples with expression levels lower than the median were assigned a score of 1, whereas those with expression levels higher than or equal to the median were assigned a score of 0. The assigned hub gene scores were subsequently used as input variables to construct the ANN model. Based on the training dataset GSE1428, the ANN model was established using the R package “neuralnet”, and its performance was validated in the GSE674 dataset. ROC curves were generated for both the training and validation datasets, and AUC values were calculated.

### 2.7. Single-Cell RNA Sequencing Analysis

Raw single-cell RNA sequencing (scRNA-seq) data were downloaded from the GSE172410 dataset, which contains single cells isolated from skeletal muscle tissues of young mice (6 months old, *n* = 3) and aged mice (24 months old, *n* = 3). Initial data preprocessing was performed using the Seurat package. After normalization, the top 1500 highly variable genes were selected using variance-stabilizing transformation for subsequent analyses. Data integration was performed using the IntegrateData function, followed by data scaling with the ScaleData function and dimensionality reduction using RunPCA. Cell clustering was then conducted using the FindNeighbors, FindClusters, and RunUMAP functions. Dot plots were generated using the DotPlot function to simultaneously display the proportion of cells expressing the core genes and their average expression levels across different cell types, thereby visualizing cell type-specific expression patterns.

### 2.8. Gene Set Enrichment Analysis

Gene set enrichment analysis (GSEA) was performed to evaluate pathways and biological function alterations in the expression datasets. The analysis was conducted using the “clusterProfiler” package, with the Molecular Signatures Database (MSigDB) gene sets “c2.cp.kegg.v2023.2.Hs.symbols.gmt” and “c5.go.v2023.2.Hs.symbols.gmt” as references. Gene sets with *p* < 0.05 were considered significantly enriched.

### 2.9. Prediction of the mRNA–miRNA–lncRNA Axis and Construction of the ceRNA Network

The miRNA targets of hub mRNAs were predicted using miRanda standalone software (v3.3a; http://www.microrna.org [accessed on 18 March 2026]), miRDB (https://mirdb.org/ [accessed on 18 March 2026]), and TargetScan (http://www.targetscan.org/vert_72/ [accessed on 18 March 2026]). The intersecting miRNAs predicted by the three databases were identified as candidate miRNAs. Subsequently, lncRNAs targeting these miRNAs were predicted using the Spongescan database (http://spongescan.rc.ufl.edu). Finally, an mRNA–miRNA–lncRNA regulatory network was constructed using Cytoscape.

### 2.10. Identification of Potential Drugs

Potential candidate drugs targeting creatine kinase, mitochondrial 2 (CKMT2), actin alpha cardiac muscle 1 (ACTC1), and forkhead box O3 (FOXO3) were predicted using the Drug Signatures Database (DSigDB) through the Enrichr platform (https://amp.pharm.mssm.edu/Enrichr/ [accessed on 25 March 2026]). Candidate drugs were ranked in ascending order according to their *p*-values, and drugs with *p* < 0.05 were considered potential drug candidates. The predicted candidate drugs and hub genes were then imported into Cytoscape 3.9.1 to construct and visualize the drug–gene interaction network.

### 2.11. Molecular Docking

The three-dimensional structures of the key candidate drugs were downloaded from the PubChem database and converted from SDF format to PDB format using Open Babel Toolkit version 3.1.1. The three-dimensional structures of the selected target proteins were obtained from the Protein Data Bank (PDB; http://www.rcsb.org/ [accessed on 18 April 2026]) or the AlphaFold Protein Structure Database. The protein identifiers used in this study were as follows: ACTC1 (PDB ID: 9BPM), CKMT2 (AlphaFold ID: AF-P17540-F1), and FOXO3 (PDB ID: 2K86). The three compounds used in this study were Alsterpaullone (PubChem CID: 5005498), Perhexiline (PubChem CID: 4746), and Wortmannin (PubChem CID: 312145). Molecular docking parameters were prepared using AutoDockTools 1.5.6, docking calculations were performed using AutoDockTools (version 1.5.6), and the docking results were visualized using PyMOL 2.4.0.

### 2.12. Molecular Dynamics Simulation

Molecular dynamics (MD) simulations were performed to evaluate the dynamic stability and conformational behavior of selected protein–ligand complexes. Based on the molecular docking results, the three complexes with the lowest binding energies, namely Alsterpaullone–FOXO3, Perhexiline–FOXO3, and Alsterpaullone–CKMT2, were selected for 100 ns MD simulations using GROMACS 2023.2. The Amber99SB-ILDN force field was applied to parameterize the protein structures, and the TIP3P water model was used for solvation. Each complex was placed in a dodecahedral simulation box, and Na^+^ and Cl^−^ ions were added to neutralize the system. Energy minimization was performed using the steepest descent algorithm until the maximum force was below 10.0 kJ/mol/nm. The systems were then equilibrated under NVT and NPT ensembles for 100 ps each at 300 K and 1.0 atm. The V-rescale thermostat and Parrinello–Rahman barostat were used for temperature and pressure coupling, respectively. A 2 fs time step was applied, covalent bonds involving hydrogen atoms were constrained using the LINCS algorithm, and long-range electrostatic interactions were treated using the particle mesh Ewald method with a cutoff of 1.0 nm. After equilibration, each system was subjected to a 100 ns production simulation. Trajectory analyses were performed using built-in GROMACS tools. Root mean square deviation (RMSD), root mean square fluctuation (RMSF), radius of gyration (Rg), and hydrogen bond analysis were used to assess conformational stability, residue-level flexibility, structural compactness, and ligand–protein interaction persistence, respectively. Free energy landscapes were further constructed based on RMSD and Rg to characterize the conformational distribution and stable energy states of the complexes.

### 2.13. Animals

Wild-type male C57BL/6J mice were purchased from Jiangsu GemPharmatech Co., Ltd. (Nanjing, China). Twenty 5-week-old male C57BL/6J mice were used to establish a natural aging model. The mice were randomly divided into a young control group (Youth, *n* = 10) and an aged group (Aged, *n* = 10). All mice were housed in a specific pathogen-free (SPF) animal facility at the Laboratory Animal Center of Shanghai University of Sport under a 12 h light/dark cycle, with the temperature maintained at 21–25 °C and humidity at 40–50%. Mice had free access to food and water. All mice were fed a standard irradiated maintenance diet for experimental mice (XTI01WC series; Jiangsu Xietong Pharmaceutical Bioengineering Co., Ltd., Jiangsu, China), in which approximately 11.1% of total energy was derived from fat according to the manufacturer’s information. Food intake was recorded daily, and body weight was measured every Friday. All animal experimental procedures were approved by the Animal Ethics Committee of Shanghai University of Sport (approval number: 102772023DW022).

### 2.14. Tissue Collection

Mice in the aged group were sacrificed at 20 months of age, whereas mice in the young group were sacrificed at 6 months of age. The mice were deeply anesthetized with 3% isoflurane and then euthanized by cervical dislocation. Bilateral gastrocnemius muscles were rapidly excised on ice using autoclaved scissors and forceps. A portion of the gastrocnemius muscle was placed in muscle fixation solution for morphological analysis, whereas the remaining tissue was snap-frozen in liquid nitrogen and stored at −80 °C for subsequent RNA extraction and gene expression analysis.

### 2.15. Cell Culture

C2C12 mouse myoblasts were purchased from the China Center for Type Culture Collection (CCTCC). Cells were cultured in Dulbecco’s modified Eagle’s medium (DMEM) supplemented with 10% fetal bovine serum (164210, Procell, Wuhan, China) and 1% penicillin–streptomycin solution (Beyotime, Shanghai, China) at 37 °C in a humidified incubator containing 5% CO_2_. When C2C12 myoblasts reached approximately 80% confluence, the culture medium was replaced with DMEM containing 2% horse serum (BL209A, Biosharp, Beijing, China) to induce myogenic differentiation. The medium was changed daily, and differentiated myotubes formed after 5–6 days were used for subsequent experiments. Successfully differentiated C2C12 myotubes were divided into a normal control group (NC) and a D-galactose-induced senescence model group (D-Gal). To establish the in vitro senescence model, C2C12 myotubes were treated with 20 mg/mL D-galactose (G5388, Sigma-Aldrich, St. Louis, MO, USA) for 48 h.

### 2.16. RNA Extraction and Gene Expression Analysis

Total RNA was extracted from gastrocnemius muscle tissues and C2C12 cells using a total RNA extraction reagent (BS258A, Biosharp, Beijing, China). RNA purity and concentration were determined by measuring absorbance at 260 and 280 nm using a Thermo Scientific NanoDrop™ One Microvolume Spectrophotometer. Complementary DNA (cDNA) was synthesized using a cDNA synthesis kit (AG11706, Accurate Biotechnology (Hunan) Co., Ltd., Changsha, China). Real-time PCR was performed using SYBR Green Premix Pro Taq HS qPCR Kit (ROX Plus) (AG11701, Accurate Biotechnology (Hunan) Co., Ltd., Changsha, China) to detect mRNA expression levels. Hprt was used as the housekeeping gene, and the relative expression levels of target genes were calculated using the 2^−ΔΔCt^ method.The primer sequences used in this study are listed in Table 2.

### 2.17. Statistical Analysis

qRT-PCR data are presented as the mean ± standard deviation (SD). Statistical analyses and graph generation were performed using GraphPad Prism 9.0. Comparisons between two groups were conducted using an independent-samples *t*-test. A value of *p* < 0.05 was considered statistically significant.

## 3. Results

### 3.1. Identification and Enrichment Analysis of DE-FAOGs

**Figure 1:**
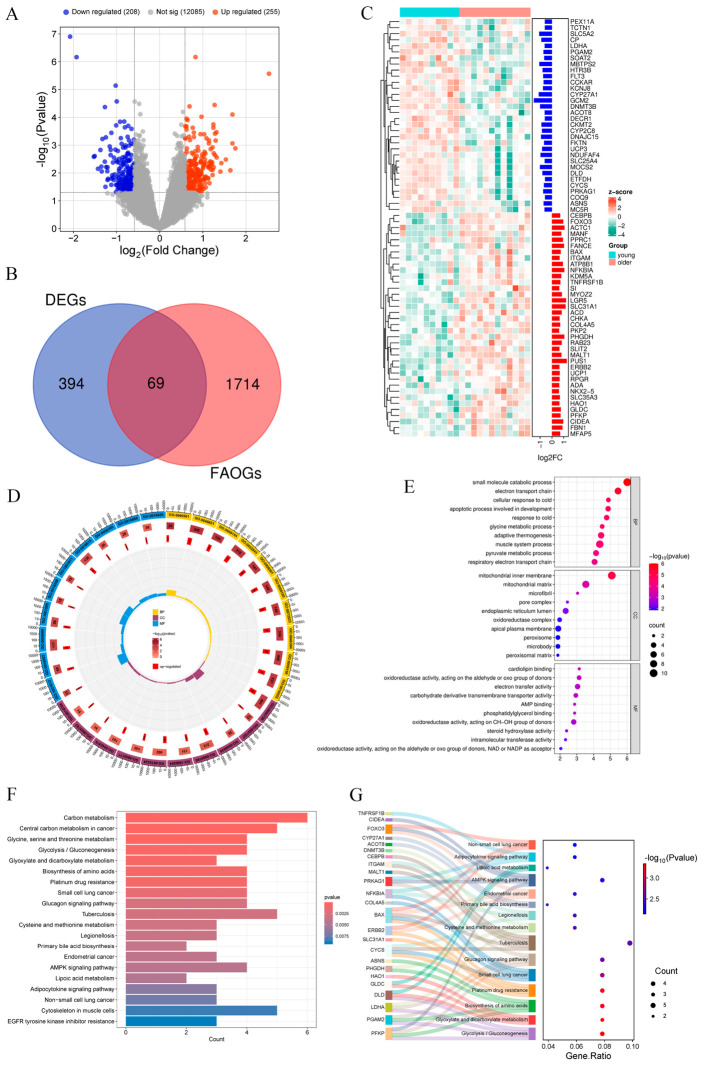
Identification and functional enrichment analysis of differentially expressed fatty acid oxidation-related genes in aged skeletal muscle. (**A**) Volcano plot of differentially expressed genes between the young and older groups in the GSE1428 dataset. (**B**) Venn diagram showing the intersection between differentially expressed genes and fatty acid oxidation-related genes (FAOGs), identifying 69 differentially expressed FAOGs. The blue circle represents DEGs, the yellow circle represents FAOGs, and their overlapping green region indicates differentially expressed FAOGs.. (**C**) Heatmap showing the expression patterns of the 69 differentially expressed FAOGs in the young and older groups. The color bar on the right represents the log2FC scale, where red indicates upregulation and blue indicates downregulation in the older group compared to the young group. (**D**) Circular plot of GO enrichment analysis of differentially expressed FAOGs. (**E**) Bubble plot of GO enrichment analysis of differentially expressed FAOGs, showing the top 10 significantly enriched terms in the BP, CC, and MF categories. (**F**) Bar plot showing the top 20 significantly enriched KEGG pathways of differentially expressed FAOGs. (**G**) Sankey diagram showing the relationships between differentially expressed FAOGs and major KEGG pathways.

Differential expression analysis was performed using the “limma” package based on transcriptomic data from the vastus lateralis muscle of young and older individuals in the training dataset GSE1428. A total of 463 differentially expressed genes (DEGs) were identified (Figure 1A). To further focus on fatty acid oxidation-related molecular alterations in aged skeletal muscle, these DEGs were intersected with 1783 FAOGs obtained from the GeneCards database. Finally, 69 DE-FAOGs were identified (Figure 1B; Supplementary Table S1). Heatmap analysis showed distinct expression patterns of these DE-FAOGs between the young and older groups, suggesting that FAO-related transcriptional programs may be involved in skeletal muscle aging (Figure 1C).

To characterize the biological functions associated with these DE-FAOGs, Gene Ontology (GO) and Kyoto Encyclopedia of Genes and Genomes (KEGG) enrichment analyses were performed. GO enrichment analysis showed that the 69 DE-FAOGs were significantly enriched in 494 biological process (BP), 30 cellular component (CC), and 59 molecular function (MF) terms (*p* < 0.05). Among them, representative BP terms included small molecule metabolic process, electron transport chain, adaptive thermogenesis, and respiratory electron transport chain. Representative CC terms were mainly related to mitochondrial inner membrane, mitochondrial matrix, oxidoreductase complex, and peroxisome. Representative MF terms included oxidoreductase activity, electron transfer activity, cardiolipin binding, and AMP binding (Figure 1D,E; Supplementary Table S2a). These results indicate that DE-FAOGs in aged skeletal muscle are primarily associated with mitochondrial structure and function, redox reactions, electron transport, and energy substrate metabolism.

KEGG enrichment analysis further revealed that the DE-FAOGs were significantly enriched in 41 signaling or metabolic pathways (*p* < 0.05). The major enriched pathways closely related to FAO and energy metabolism included carbon metabolism, glycolysis/gluconeogenesis, AMPK signaling pathway, and adipocytokine signaling pathway (Figure 1F,G; Supplementary Table S2b). These pathways are closely associated with cellular energy metabolism, substrate utilization, lipid metabolic regulation, and mitochondrial metabolic remodeling, suggesting that FAO abnormalities in aged skeletal muscle may not be isolated events but may occur together with broader disturbances in the energy metabolism network during aging-related metabolic decline.

### 3.2. Identification and Validation of Hub Genes

**Figure 2:**
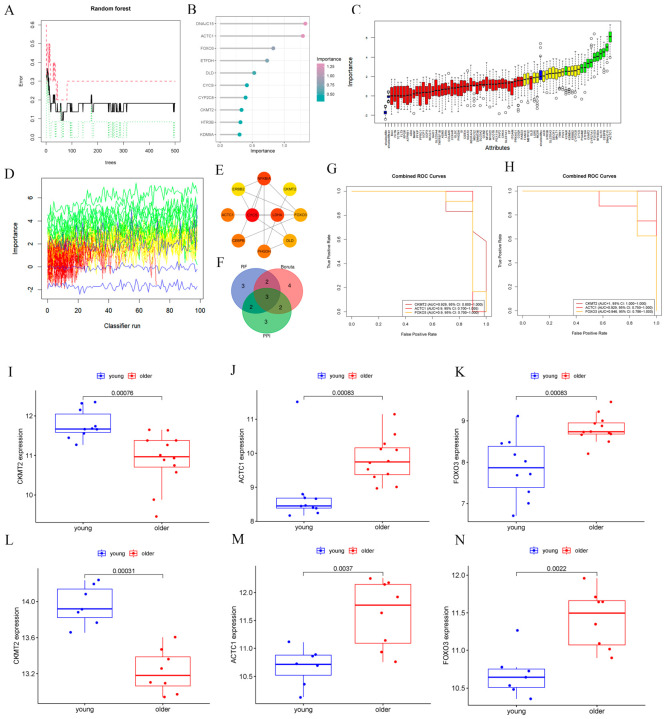
Screening and validation of hub genes associated with impaired fatty acid oxidation in aged skeletal muscle. (**A**,**B**) Candidate feature genes screened from DE-FAOGs using the random forest algorithm. In Figure A, the black solid line represents the overall out-of-bag error. The red dashed line indicates the classification error of class 0. The green dashed line shows the classification error of class 1. (**C**,**D**) Candidate genes with stable discriminatory ability identified using the Boruta algorithm. Different bar colors separate features based on importance levels. Blue corresponds to features with the lowest importance. Red stands for features with low importance. Yellow represents features with moderate importance. Green marks features with high importance. (**E**) PPI network constructed using the STRING database and Cytoscape, showing the top 10 candidate core genes ranked by Degree values. Node colors indicate gene degree values in the protein-protein interaction network. Nodes turn red with higher degree values and yellow with lower degree values. (**F**) Intersection of the results from random forest, Boruta, and PPI network analyses, identifying CKMT2, ACTC1, and FOXO3 as consensus hub genes. Blue, red and green regions stand for hub genes screened by random forest, Boruta algorithm and protein-protein interaction network, respectively. Overlapping colored areas denote intersecting hub genes from multiple screening methods. (**G**) ROC curve analysis of CKMT2, ACTC1, and FOXO3 in the training dataset GSE1428. (**H**) ROC curve analysis of CKMT2, ACTC1, and FOXO3 in the validation dataset GSE674. (**I**–**K**) Expression levels of *CKMT2*, *ACTC1*, and *FOXO3* in the training dataset GSE1428. (**L**–**N**) Expression levels of *CKMT2*, *ACTC1*, and *FOXO3* in the validation dataset GSE674.

To identify key genes associated with impaired FAO in aged skeletal muscle from the 69 DE-FAOGs, random forest, Boruta, and PPI network analyses were performed. The random forest algorithm identified the top 10 candidate feature genes (Figure 2A,B; Supplementary Table S3a), whereas the Boruta algorithm identified 11 important candidate genes with stable discriminatory ability (Figure 2C,D; Supplementary Table S3b). In addition, PPI network analysis identified the top 10 candidate core genes according to Degree values (Figure 2E; Supplementary Table S3c). By intersecting the results obtained from these three approaches, *CKMT2*, *ACTC1*, and *FOXO3* were identified as common hub genes associated with impaired FAO in aged skeletal muscle (Figure 2F; Supplementary Table S3d). Among them, *CKMT2* was downregulated in the older group, whereas *ACTC1* and *FOXO3* were upregulated.

ROC curve analysis was subsequently performed to evaluate the discriminatory ability of the three hub genes. In the training dataset GSE1428, the AUC values of CKMT2, ACTC1, and FOXO3 were 0.929, 0.900, and 0.900, respectively (Figure 2G). In the validation dataset GSE674, the corresponding AUC values were 1.000, 0.929, and 0.946, respectively (Figure 2H), indicating good performance in distinguishing skeletal muscle aging status. Further expression validation showed that *CKMT2* was significantly downregulated in the older group, whereas *ACTC1* and *FOXO3* were significantly upregulated. This expression pattern was consistent in both the training and validation datasets (Figure 2I–N). These findings suggest that *CKMT2*, *ACTC1*, and *FOXO3* may serve as potential biomarkers associated with impaired FAO in aged skeletal muscle.

### 3.3. Construction and Validation of the ANN Model

**Figure 3:**
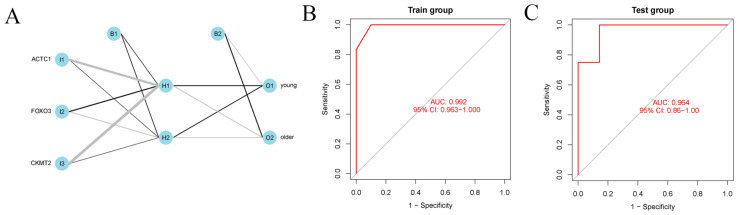
Construction and validation of the ANN diagnostic model based on hub genes. (**A**) Structure of the artificial neural network model constructed based on CKMT2, ACTC1, and FOXO3, including the input layer, hidden layer, and output layer. (**B**) ROC curve of the ANN model in the training dataset GSE1428, with an AUC of 0.992 (95% CI: 0.963–1.000). (**C**) ROC curve of the ANN model in the validation dataset GSE674, with an AUC of 0.964 (95% CI: 0.860–1.000).

To further evaluate the potential of CKMT2, ACTC1, and FOXO3 in combination for identifying skeletal muscle aging status, an ANN diagnostic model was constructed based on the expression features of the three hub genes. Hub gene scores were used as input variables; a hidden layer containing two nodes was set, and the classification outcome of the young and older groups was used as the output variable to construct the ANN classification model (Figure 3A).

ROC curve analysis showed that the ANN model exhibited strong discriminatory performance in the training dataset GSE1428, with an AUC value of 0.992 (95% CI: 0.963–1.000) (Figure 3B). Further validation in the external validation dataset GSE674 yielded an AUC value of 0.964 (95% CI: 0.860–1.000) (Figure 3C). These results indicate that the ANN model based on CKMT2, ACTC1, and FOXO3 effectively distinguished the young and older groups, suggesting that the combined use of these three hub genes may have good predictive value for skeletal muscle aging status.

### 3.4. Single-Cell RNA Sequencing Analysis

**Figure 4:**
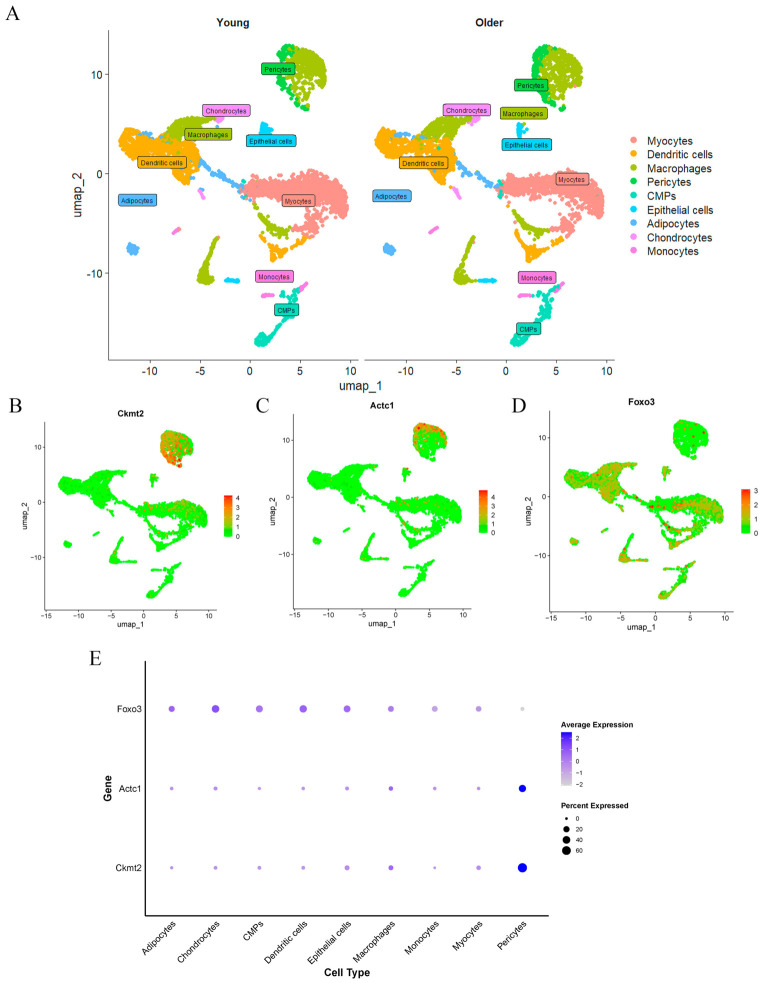
Expression distribution of hub genes in the single-cell atlas of aged skeletal muscle. (**A**) UMAP plot of skeletal muscle cells from young and aged groups based on the GSE172410 dataset. (**B**–**D**) UMAP feature plots showing the expression distribution of *Ckmt2*, *Actc1*, and *Foxo3* across different cell populations. (**E**) Dot plot showing the proportion of cells expressing Ckmt2, Actc1, and Foxo3 and their average expression levels across different cell types.

To further clarify the expression distribution of CKMT2, ACTC1, and FOXO3 across different cell populations in aged skeletal muscle, single-cell RNA sequencing (scRNA-seq) analysis was performed using the GSE172410 dataset. After quality control, data integration, dimensionality reduction, clustering, and cell type annotation, nine major cell types were identified in skeletal muscle samples from the young and aged groups, including myocytes, dendritic cells, macrophages, pericytes, common myeloid progenitors (CMPs), epithelial cells, adipocytes, chondrocytes, and monocytes (Figure 4A). The expression distribution of the three hub genes was then analyzed across different cell populations. The results showed that *Ckmt2* and *Actc1* exhibited relatively higher expression in pericytes and macrophages, whereas *Foxo3* showed relatively higher expression in chondrocytes and dendritic cells (Figure 4B–E). These findings suggest that CKMT2, ACTC1, and FOXO3 may exhibit distinct cell population-biased expression patterns within the aged skeletal muscle microenvironment, reflecting their potential involvement in multicellular regulatory processes associated with metabolic cells, immune cells, and stromal cells during skeletal muscle aging.

### 3.5. GSEA and Construction of the Predicted ceRNA Network

**Figure 5:**
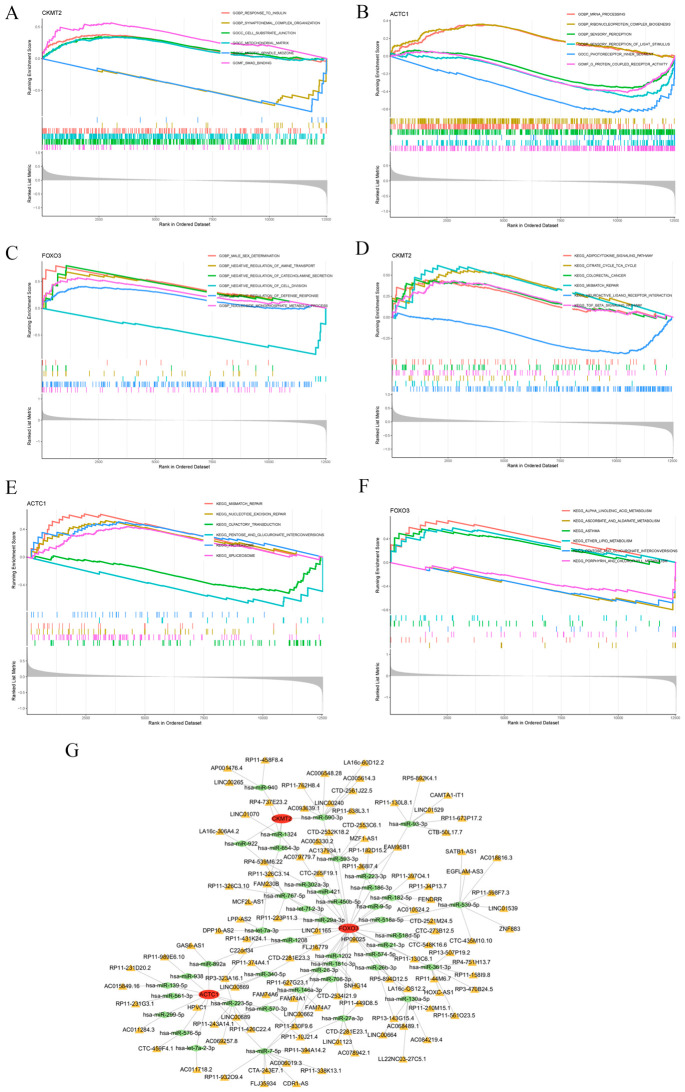
GSEA of hub genes and construction of the predicted mRNA–miRNA–lncRNA ceRNA network. (**A**–**C**) GO-GSEA results associated with CKMT2, ACTC1, and FOXO3. (**D**–**F**) KEGG-GSEA results associated with CKMT2, ACTC1, and FOXO3. (**G**) Predicted mRNA–miRNA–lncRNA ceRNA regulatory network constructed based on CKMT2, *ACTC1*, and *FOXO3*. Red nodes represent hub genes, green nodes represent miRNAs, and orange nodes represent lncRNAs.

To further explore the potential biological functions associated with CKMT2, ACTC1, and FOXO3, GO- and KEGG-based GSEA analyses were performed for each hub gene. The GO-GSEA results showed that CKMT2-related gene sets were mainly enriched in terms such as response to insulin, mitochondrial matrix, cell–substrate junction, and SMAD binding (Figure 5A), suggesting that CKMT2 may be associated with insulin response, mitochondrial function, and cellular structural regulation in aged skeletal muscle. ACTC1-related gene sets were mainly involved in mRNA processing, ribonucleoprotein complex biogenesis, sensory perception, and G protein-coupled receptor activity (Figure 5B), suggesting that ACTC1 may be related to RNA processing, ribonucleoprotein complex biogenesis, and signal perception-related processes. FOXO3-related gene sets were mainly enriched in negative regulation of amine transport, negative regulation of catecholamine secretion, negative regulation of cell division, negative regulation of defense response, and nucleoside monophosphate metabolic process (Figure 5C), indicating that FOXO3 may participate in cell cycle suppression, stress-defense regulation, and nucleotide metabolism in aged skeletal muscle.

KEGG-GSEA further showed that CKMT2-related pathways mainly included the adipocytokine signaling pathway, citrate cycle/TCA cycle, mismatch repair, and TGF-β signaling pathway (Figure 5D), suggesting that CKMT2 may be associated with energy metabolism, adipokine signaling, and tissue remodeling-related processes. ACTC1-related pathways mainly involved mismatch repair, nucleotide excision repair, pentose and glucuronate interconversions, proteasome, and spliceosome (Figure 5E), indicating that ACTC1 may be related to DNA damage repair, protein homeostasis, and RNA splicing. FOXO3-related pathways were mainly enriched in alpha-linolenic acid metabolism, ether lipid metabolism, pentose and glucuronate interconversions, ascorbate and aldarate metabolism, and porphyrin and chlorophyll metabolism (Figure 5F), suggesting that FOXO3 may be associated with lipid metabolism, redox metabolism, and carbohydrate derivative metabolism. Overall, the GSEA results indicate that the three hub genes are not only associated with FAO-related metabolic abnormalities but may also participate in multidimensional pathological processes in aged skeletal muscle, including mitochondrial function, lipid metabolism, insulin response, cellular stress, and tissue remodeling.

In addition, to predict the potential post-transcriptional regulatory mechanisms of the hub genes, an mRNA–miRNA–lncRNA-predicted ceRNA network was constructed. By integrating the prediction results from the miRanda, miRDB, and TargetScan databases, 44 candidate miRNAs associated with *CKMT2*, *ACTC1*, and *FOXO3* were identified. Subsequently, 110 potential lncRNAs were predicted using the Spongescan database, and an mRNA–miRNA–lncRNA regulatory network was constructed using Cytoscape (Figure 5G; Supplementary Table S4a,b). This network suggests that *CKMT2*, *ACTC1*, and *FOXO3* may be jointly regulated by multiple miRNAs and lncRNAs, providing candidate directions for further investigation of upstream noncoding RNA-mediated regulatory mechanisms underlying impaired FAO in aged skeletal muscle.

### 3.6. Drug–Gene Interaction and Molecular Docking Analysis

**Figure 6:**
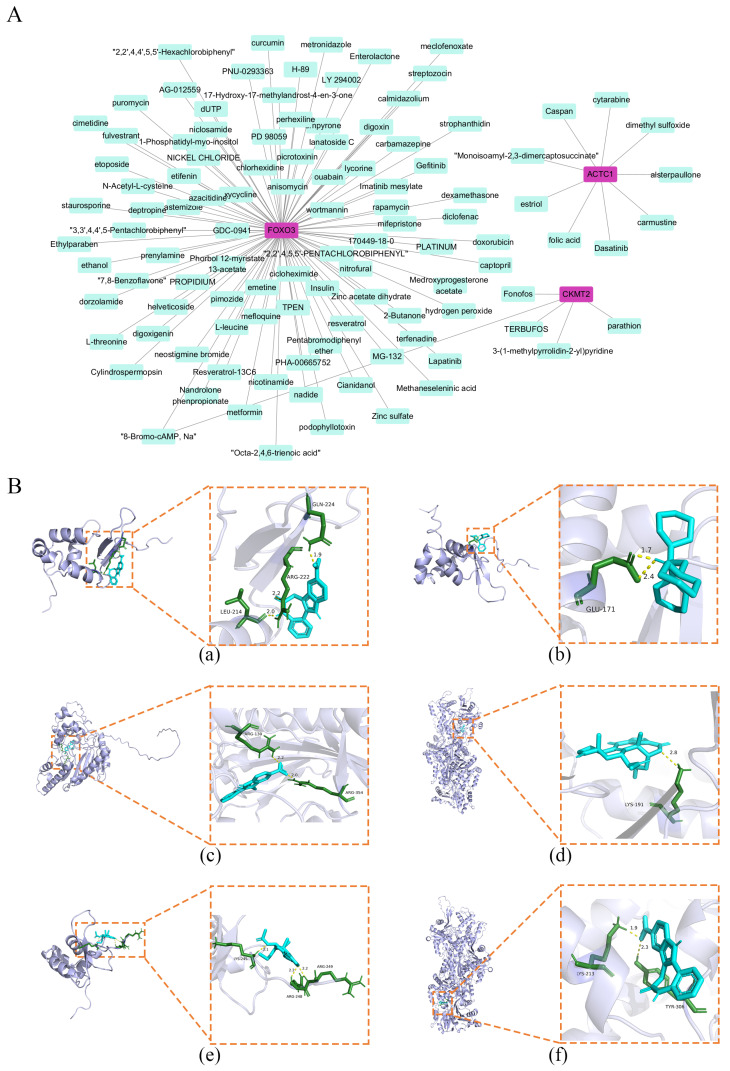
Drug–gene interaction and molecular docking analysis related to hub genes. (**A**) Interaction network between hub genes and candidate drugs. Purple rectangles represent hub genes, and blue rectangles represent candidate drugs. (**B**) Detailed views of molecular docking results: (**a**) Alsterpaullone–FOXO3; (**b**) Perhexiline–FOXO3; (**c**) Alsterpaullone–CKMT2; (**d**) Wortmannin–ACTC1; (**e**) Wortmannin–FOXO3; (**f**) Alsterpaullone–ACTC1. The lightblue structure represents the target protein. The cyan structure corresponds to the small-molecule ligand. The green structures stand for amino acid residues involved in docking. Yellow dashed lines mark hydrogen bonds, and the numbers indicate hydrogen bond lengths in angstroms.

To further explore potential pharmacological intervention clues for CKMT2, ACTC1, and FOXO3, candidate drugs targeting the three hub genes were predicted using the DSigDB database. Using *p* < 0.05 as the screening criterion, a series of potential candidate drugs associated with the hub genes were identified (Supplementary Table S5). The predicted interactions between candidate drugs and hub genes were then imported into Cytoscape for visualization, and a drug–gene interaction network was constructed (Figure 6A). Based on the drug prediction results, Alsterpaullone, Perhexiline, and Wortmannin were further selected as representative candidate drugs and subjected to molecular docking analysis with CKMT2, ACTC1, and FOXO3. The results showed that all three candidate drugs exhibited potential binding affinity with the proteins encoded by the three hub genes, with predicted binding energies below −5.0 kcal/mol (Table 3). Among them, six drug–protein complexes with relatively low binding energies and reasonable docking conformations were further visualized, including Alsterpaullone–FOXO3, Perhexiline–FOXO3, Alsterpaullone–CKMT2, Wortmannin–ACTC1, Wortmannin–FOXO3, and Alsterpaullone–ACTC1 (Figure 6B).

### 3.7. Molecular Dynamics Simulation

**Figure 7:**
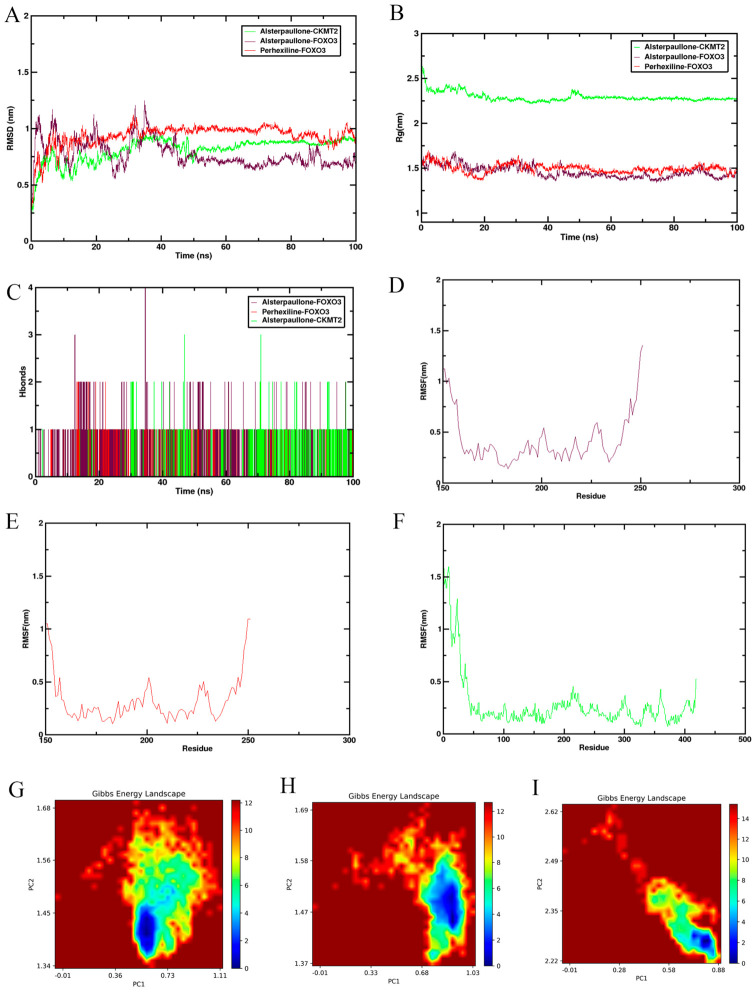
Dynamic stability analysis of protein–ligand complexes during 100 ns molecular dynamics simulations. (**A**) RMSD curves of the Alsterpaullone–FOXO3, Perhexiline–FOXO3, and Alsterpaullone–CKMT2 complexes. (**B**) Rg curves of the three complexes. (**C**) Changes in the number of hydrogen bonds during the simulations of the three complexes. (**D**–**F**) RMSF curves of the three complexes. The purple, red and green lines represent the RMSF curves of the Alsterpaullone-FOXO3, Perhexiline-FOXO3, and Alsterpaullone-CKMT2 complexes, respectively. (**G**–**I**) Free energy landscapes of the Alsterpaullone–FOXO3, Perhexiline–FOXO3, and Alsterpaullone–CKMT2 complexes.

To further evaluate the conformational stability of candidate drug–protein complexes in a dynamic environment, 100 ns molecular dynamics (MD) simulations were performed for the Alsterpaullone–FOXO3, Perhexiline–FOXO3, and Alsterpaullone–CKMT2 complexes, which showed relatively low binding energies in the molecular docking analysis. RMSD was used to quantify the deviation of atomic coordinates from the initial structure and is a reliable indicator for evaluating system stability [19]. RMSD analysis was first performed to assess the overall conformational deviation of the complexes. The results showed that the Alsterpaullone–FOXO3 complex exhibited fluctuations during 0–50 ns and then gradually reached equilibrium, with a stable-stage RMSD of approximately 7.3 Å. The Perhexiline–FOXO3 complex underwent conformational adjustment during 0–35 ns and subsequently reached a relatively stable state, with an RMSD plateau of approximately 10 Å, suggesting evident conformational adaptation during the simulation. The Alsterpaullone–CKMT2 complex gradually stabilized during 0–60 ns, and its RMSD was maintained at approximately 8.8 Å in the later stage, indicating relatively good overall conformational stability (Figure 7A).

Rg analysis provides a measure of the overall compactness and size of a complex and offers insights into conformational changes during complex formation [19]. Larger Rg values generally indicate more flexible or expanded complexes, whereas smaller Rg values reflect more compact and rigid structures [19]. Therefore, Rg and hydrogen bond analyses were further performed to evaluate the overall compactness and interaction stability of the complexes. Rg analysis showed that the Alsterpaullone–FOXO3, Perhexiline–FOXO3, and Alsterpaullone–CKMT2 complexes stabilized at approximately 14 Å, 15 Å, and 23 Å, respectively, during the later stages of simulation, indicating that their overall structures remained relatively stable throughout the simulations (Figure 7B). Hydrogen bond analysis showed that all three complexes formed a certain number of hydrogen bonds during the simulation, with the number of hydrogen bonds mainly fluctuating within the ranges of 0–4, 0–2, and 0–3, respectively (Figure 7C).

RMSF analysis is an important tool for evaluating subtle changes in protein chains during simulation and reflects residue-level fluctuations [19]. RMSF analysis was therefore performed to assess the local flexibility of protein residues during the simulation. The average RMSF values of the Alsterpaullone–FOXO3, Perhexiline–FOXO3, and Alsterpaullone–CKMT2 complexes were approximately 4.3 Å, 3.4 Å, and 2.8 Å, respectively, indicating different degrees of local conformational fluctuation among the complexes (Figure 7D–F). Finally, free energy landscapes were constructed based on RMSD and Rg. The results showed that all three complexes formed relatively concentrated low-energy regions, suggesting that they reached relatively stable conformational states during the simulation (Figure 7G–I). Overall, the MD simulation results support a certain degree of dynamic stability for the Alsterpaullone–FOXO3, Perhexiline–FOXO3, and Alsterpaullone–CKMT2 complexes.

### 3.8. qRT-PCR Validation of Differentially Expressed Genes

**Figure 8:**
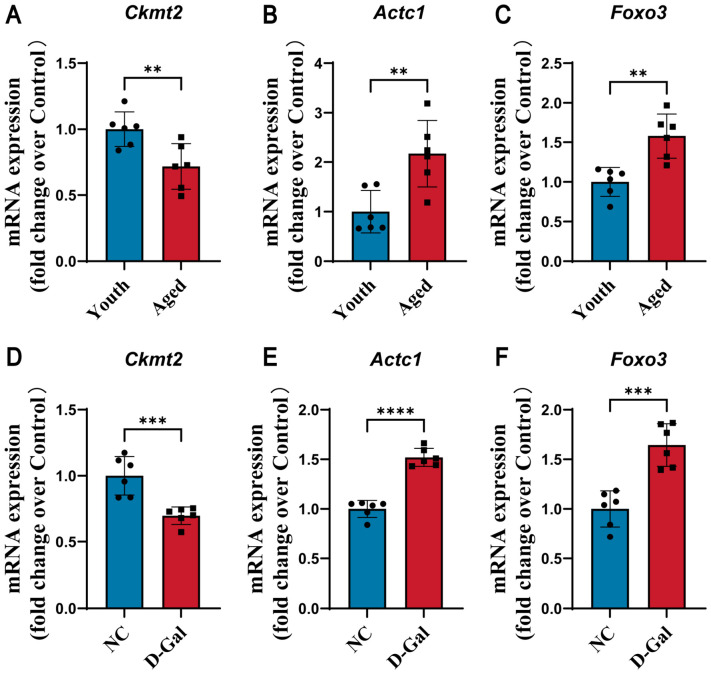
qRT-PCR validation of hub gene expression changes in in vivo and in vitro aging models. (**A**–**C**) mRNA expression levels of *Ckmt2*, *Actc1*, and *Foxo3* in gastrocnemius muscle tissues from young and aged mice (*n* = 6 per group). (**D**–**F**) mRNA expression levels of *Ckmt2*, *Actc1*, and *Foxo3* in control and D-galactose-treated C2C12 myotubes (*n* = 6 per group). Data are presented as the mean ± SD. ** *p* < 0.01, *** *p* < 0.001, **** *p* < 0.0001.

Finally, the mRNA expression levels of Ckmt2, Actc1, and Foxo3 were examined in naturally aged mouse skeletal muscle tissues and an in vitro cellular senescence model to validate the actual expression changes in the hub genes identified by bioinformatics and machine learning analyses. First, qRT-PCR was performed using gastrocnemius muscle tissues from 6-month-old young mice and 20-month-old aged mice. Compared with the young group, *Ckmt2* expression was significantly downregulated, whereas *Actc1* and *Foxo3* expression levels were significantly upregulated in the gastrocnemius muscles of aged mice (Figure 8A–C). This expression pattern was consistent with the transcriptomic results obtained from both the training and validation datasets, suggesting that *Ckmt2*, *Actc1*, and *Foxo3* exhibit stable expression patterns in naturally aged skeletal muscle. To further validate the expression changes in these hub genes in an in vitro aging model, differentiated C2C12 myotubes were treated with D-galactose to establish a cellular senescence model, and the expression levels of *Ckmt2*, *Actc1*, and *Foxo3* were measured. Compared with the control group, D-galactose-treated C2C12 myotubes showed decreased *Ckmt2* expression and increased *Actc1* and *Foxo3* expression (Figure 8D–F). These results demonstrate that *Ckmt2*, *Actc1*, and *Foxo3* showed expression trends consistent with those observed in public transcriptomic datasets in both in vivo and in vitro aging models, further supporting their reliability as potential biomarkers associated with impaired FAO in aged skeletal muscle.

## 4. Discussion

In 1989, Rosenberg [20] first coined the term “sarcopenia” to describe the age-related decline in skeletal muscle mass. With advances in research, sarcopenia is no longer regarded merely as a reduction in muscle mass but is now recognized as an aging-related muscle disease characterized by declines in muscle strength, muscle quantity, and physical performance [21]. Its clinical significance lies in its direct impairment of mobility, independence in daily living, and physiological resilience in older adults [21]. A large body of epidemiological and cohort evidence has shown that sarcopenia is closely associated with adverse outcomes, including falls, fractures, hospitalization, and disability [22,23]. In addition, sarcopenic status is associated with an increased risk of all-cause mortality, indicating that sarcopenia is an important pathological condition affecting overall health outcomes and long-term survival in older adults [24]. Skeletal muscle is a major tissue responsible for fatty acid uptake, storage, and oxidative utilization. FAO is closely coupled with the mitochondrial tricarboxylic acid (TCA) cycle, oxidative phosphorylation, and ATP production, thereby maintaining muscle contraction, substrate switching, and metabolic flexibility. During aging, skeletal muscle commonly exhibits mitochondrial dysfunction, increased intramyocellular lipid accumulation, and reduced metabolic flexibility, ultimately contributing to lipotoxicity and skeletal muscle functional decline [25,26,27]. In the context of aging, sarcopenia often coexists with increased fat mass, resulting in the so-called sarcopenic obesity phenotype [28]. This condition is characterized not only by reduced skeletal muscle mass and strength but also by visceral fat accumulation, intermuscular fat infiltration, chronic low-grade inflammation, and insulin resistance, which further aggravate the disruption of skeletal muscle metabolic homeostasis [28]. For skeletal muscle, increased fatty acid supply does not necessarily indicate enhanced oxidative utilization. When mitochondrial FAO capacity declines, excess lipids are more likely to be converted into lipid intermediates, such as diacylglycerols, ceramides, and acylcarnitines, thereby interfering with insulin signaling, promoting inflammatory responses, and exacerbating mitochondrial damage [29,30]. Therefore, sarcopenic obesity may represent a specific aging-related metabolic state characterized by excessive lipid supply but insufficient oxidative disposal. Moreover, muscle biopsy studies in older adults have confirmed that patients with sarcopenia exhibit reduced mitochondrial content, function, and energetic efficiency in skeletal muscle, which may further restrict the effective coupling between fatty acid β-oxidation and oxidative phosphorylation, thereby aggravating ATP insufficiency, muscle fatigue, and functional decline [31]. These findings suggest that impaired FAO is not only an important manifestation of skeletal muscle metabolic dysfunction during aging and sarcopenia but may also contribute to sarcopenia progression by promoting lipotoxicity, mitochondrial dysfunction, and loss of metabolic flexibility. Therefore, identifying biomarkers of aged skeletal muscle from the perspective of FAO abnormalities is of clear practical and translational significance for elucidating the molecular mechanisms underlying sarcopenia and for discovering potential biomarkers that reflect age-related skeletal muscle functional decline. In this context, the three-gene signature consisting of *CKMT2*, *ACTC1*, and *FOXO3* identified in this study may not only reflect FAO abnormalities in aged skeletal muscle but also provide candidate molecular clues for identifying.

Mitochondrial quantity, density, and quality are fundamental determinants of skeletal muscle fatty acid oxidation capacity. With advancing age, skeletal muscle often exhibits reduced mitochondrial content, disrupted cristae structure, weakened respiratory chain complex function, and impaired mitochondrial quality control [32,33]. These alterations can weaken the effective coupling between fatty acid β-oxidation and oxidative phosphorylation, thereby reducing the efficiency of fatty acids as energy substrates [32,33]. Meanwhile, aging-related declines in mitochondrial respiratory efficiency and dysfunction of the electron transport chain may increase electron leakage and reactive oxygen species (ROS) production, thereby inducing oxidative stress [34]. Excessive ROS can further damage mitochondrial membrane structures, respiratory chain complexes, and key metabolic enzymes, while aggravating lipid peroxidation and mitochondrial metabolic burden. This may form a vicious cycle that collectively promotes metabolic dysfunction and functional decline in aged skeletal muscle [35]. In the present study, DE-FAOGs were enriched in terms such as electron transport chain, oxidoreductase complex, oxidoreductase activity, and electron transfer activity, also suggesting that redox imbalance and mitochondrial electron transport abnormalities may participate in FAO-related metabolic remodeling in aged skeletal muscle. Conversely, aerobic exercise and endurance training are considered effective non-pharmacological interventions for improving mitochondrial function and metabolic flexibility in aged skeletal muscle. Exercise training can activate AMPK–PGC-1α, PPAR signaling, and mitochondrial biogenesis-related pathways, thereby enhancing skeletal muscle oxidative metabolic capacity, promoting fatty acid uptake, transport, and mitochondrial oxidative utilization, and improving lipid deposition, oxidative stress, and insulin sensitivity [36,37]. Therefore, changes in the expression of FAO-related hub genes may also serve as candidate molecular indicators for evaluating the effects of exercise training, nutritional intervention, or metabolic intervention on improving energy metabolism in aged skeletal muscle. However, this study did not directly measure ROS production, lipid peroxidation, mitochondrial density, mitochondrial respiratory function, or FAO flux; therefore, these mechanisms require further validation in future studies.

In this study, 69 differentially expressed FAO-related genes were first identified from transcriptomic data of human vastus lateralis muscle. These genes were mainly enriched in pathways and cellular components related to the mitochondrial inner membrane, mitochondrial matrix, electron transport chain, carbon metabolism, and glycolysis/gluconeogenesis. These findings suggest that FAO abnormalities in aged skeletal muscle are not isolated events but are accompanied by impaired mitochondrial oxidative phosphorylation and broad remodeling of the energy metabolism network. Accumulating evidence indicates that reduced mitochondrial content, decreased respiratory chain efficiency, and impaired metabolic flexibility are important drivers of skeletal muscle functional decline during aging [25,38]. FAO is highly coupled with the TCA cycle, oxidative phosphorylation, and ATP production. Impaired FAO not only reduces fatty acid utilization efficiency but also promotes the accumulation of lipid intermediates, enhances oxidative stress, and decreases skeletal muscle insulin sensitivity [7,14]. Therefore, the mitochondrial and energy metabolism-related signals revealed by the enrichment analyses in this study are highly consistent with current understanding of metabolic remodeling in aged skeletal muscle and further support impaired FAO as an important metabolic feature of skeletal muscle aging.

By integrating random forest, Boruta, and PPI network analyses, this study identified *CKMT2*, *ACTC1*, and *FOXO3* as three hub genes. Notably, these three genes correspond to distinct biological dimensions, including mitochondrial energy metabolism, muscle fiber structural remodeling, and stress regulation, suggesting that FAO abnormalities in aged skeletal muscle may involve coordinated dysregulation of metabolic, structural, and transcriptional regulatory networks. CKMT2 encodes mitochondrial creatine kinase and is an important component of the phosphocreatine shuttle system. It transfers high-energy phosphate groups generated in mitochondria to the cytosol, thereby supporting rapid ATP supply in skeletal muscle and playing an essential role in maintaining energy metabolic homeostasis and mitochondrial function [39]. From the perspective of metabolic coupling, skeletal muscle FAO generates acetyl-CoA and NADH/FADH_2_ through β-oxidation, which are further fed into the TCA cycle and oxidative phosphorylation for ATP production. CKMT2 is located at a key node linking mitochondrial ATP output to the phosphocreatine shuttle system and can transfer high-energy phosphate groups derived from mitochondrial oxidative metabolism to cytosolic contractile machinery [39]. Therefore, the key role of CKMT2 is to convert mitochondria-derived energy into ATP buffering capacity that is readily available for muscle contraction. Recent functional studies have also shown that reduced CKMT2 directly impairs mitochondrial respiration, membrane potential, and oxidative metabolism in skeletal muscle cells [40]. Further evidence indicates that enhancing CKMT2 expression in aged mice can significantly improve skeletal muscle mitochondrial function and exercise endurance [41]. Taken together, although CKMT2 is not a canonical fatty acid oxidation enzyme, FAO-derived energy ultimately needs to be delivered to contractile machinery through mitochondrial energy transfer systems. Thus, CKMT2 downregulation may reflect reduced coupling efficiency between FAO and oxidative phosphorylation, as well as impaired ATP buffering capacity. Recent studies have also proposed Ckmt2 as a plasma biomarker for assessing the severity of reperfusion injury after acute myocardial infarction [42], further supporting its reliability as a molecular biomarker. ACTC1 encodes a member of the α-actin family and is an important component of sarcomeric structure and the cytoskeleton [43]. Previous studies have suggested that ACTC1 can be expressed in regenerating skeletal muscle fibers and participate in the regulation of myogenic cell differentiation, making it one of the marker genes associated with muscle fiber remodeling [44]. In addition, previous evidence has shown that cardiac α-actin and skeletal muscle α-actin can be coexpressed to some extent in adult human heart and skeletal muscle [45], and ACTC1 is closely associated with abnormal cardiac contractile structures, dilated cardiomyopathy, hypertrophic cardiomyopathy, and other structural heart diseases [46,47]. Therefore, although ACTC1 cannot currently be regarded as an established skeletal muscle aging- or sarcopenia-specific biomarker, its disease relevance in striated muscle contractile structures and cardiovascular diseases suggests that altered ACTC1 expression may have potential value in reflecting sarcomeric remodeling and contractile system adaptation. In the skeletal muscle transcriptomic context of the present study, the upregulation of ACTC1 may mainly reflect sarcomeric structural remodeling, cytoskeletal adaptation, and regeneration- or stress-related structural changes in aged skeletal muscle, rather than directly representing FAO enzyme activity itself. In contrast, FOXO3 has a more direct biological connection with FAO. FOXO3 belongs to the Forkhead box O transcription factor family and is a key transcriptional regulator involved in cellular stress responses, catabolic metabolism, mitochondrial homeostasis, autophagy, apoptosis, and lifespan regulation [48]. Previous studies have shown that FOXO3 activation can promote metabolic adaptation to energy stress and nutrient deprivation, whereas sustained overactivation may promote protein degradation and muscle atrophy programs [49,50,51]. In skeletal muscle, FOXO3 can induce the expression of Atrogin-1/F-box protein 32 (FBXO32), muscle RING-finger protein 1 (MuRF1)/tripartite motif-containing 63 (TRIM63), BCL2-interacting protein 3 (BNIP3), and microtubule-associated protein 1 light chain 3 (LC3), thereby promoting protein degradation and autophagy; it is therefore considered an important regulatory node in muscle atrophy and aging [52,53]. Interestingly, recent evidence indicates that, in addition to regulating autophagy and mitophagy, FOXO3 can promote FAO by activating peroxisome proliferator-activated receptor α (PPARα) and the transcription of downstream FAO-related genes [54]. Moreover, FOXO3 has been investigated as a disease-associated molecule or potential biomarker in several non-skeletal-muscle pathological contexts. For example, FOXO3 dysregulation is associated with tumorigenesis, tumor suppression, apoptosis, and therapeutic responses, and changes in its expression or activity may have potential prognostic or biological significance in multiple cancers [55]. FOXO3 has also been proposed as a potential biomarker and therapeutic target for premature ovarian insufficiency [56]. In addition, FOXO3 may serve as a potential diagnostic biomarker related to autophagy in rheumatoid arthritis [57]. Therefore, the upregulation of FOXO3 observed in aged skeletal muscle in this study may, on the one hand, reflect a compensatory adaptive response to impaired mitochondrial oxidative metabolism and FAO. On the other hand, sustained FOXO3 activation may promote protein degradation and muscle atrophy programs, thereby contributing to sarcopenia progression. Thus, in this study, FOXO3 upregulation can be interpreted as a transcriptional regulatory node linking energy stress, protein degradation, autophagy, mitochondrial homeostasis, oxidative stress defense, and FAO-related metabolic adaptation in aged skeletal muscle.

From the perspective of biomarker utility, these three genes should be interpreted within the biological context of aged skeletal muscle rather than as isolated diagnostic indicators. The three-gene ANN model constructed in this study provides a potential strategy for improving biomarker robustness. Specifically, CKMT2 mainly reflects mitochondrial energy transfer and phosphocreatine-mediated energy buffering capacity, ACTC1 may reflect sarcomeric and contractile structural remodeling, whereas FOXO3 represents stress-related transcriptional regulation, protein homeostasis, and metabolic adaptation. Thus, these three genes cover three distinct but interrelated biological dimensions involved in impaired FAO in aged skeletal muscle, namely “energy transfer–structural remodeling–stress regulation”. Therefore, compared with a single-gene indicator, the combined model based on CKMT2, ACTC1, and FOXO3 may be more suitable for reflecting the integrated molecular alterations associated with impaired FAO in aged skeletal muscle. It should be emphasized that this study does not define ACTC1 or FOXO3 as established sarcopenia-specific diagnostic biomarkers, but rather as candidate molecules with potential stratification value in the context of skeletal muscle aging and FAO abnormalities.

However, the tissue specificity of these genes should be carefully considered when they are evaluated as potential clinical biomarkers. CKMT2 and ACTC1 are not exclusively expressed in skeletal muscle; both are also expressed in cardiac tissue. In particular, ACTC1 has been extensively studied in the context of abnormal cardiac contractile structures, hypertrophic cardiomyopathy, dilated cardiomyopathy, and other cardiovascular diseases [46,47]. In addition, CKMT2, as a member of the mitochondrial creatine kinase family, plays important roles in energy metabolism in both cardiac and skeletal muscle [39]. Therefore, if CKMT2 or ACTC1 is evaluated as a circulating biomarker, cardiovascular comorbidities, myocardial injury, and cardiac remodeling should be considered potential confounding factors. Future population-based studies should record or adjust for coronary heart disease, heart failure, cardiomyopathy, history of myocardial infarction, and cardiac-related indicators such as cardiac troponin, creatine kinase-MB (CK-MB), and N-terminal pro-B-type natriuretic peptide (NT-proBNP) to improve the interpretability of skeletal muscle-derived signals. Similarly, FOXO3 is a broadly expressed stress-responsive transcription factor and is not specific to skeletal muscle. In addition to its involvement in cancer and aging-related processes, members of the FOXO family also participate in bone metabolic homeostasis, oxidative stress defense, and the balance between bone formation and bone resorption [58]. Moreover, osteoporosis and sarcopenia frequently coexist in older adults and may form an osteosarcopenia phenotype, which further complicates the interpretation of circulating FOXO3 signals [59]. Therefore, circulating FOXO3 alone may not distinguish whether the signal originates from skeletal muscle, bone, or other tissues. Based on these considerations, the three genes identified in this study are more appropriately regarded as a skeletal muscle context-dependent candidate biomarker signature rather than stand-alone circulating diagnostic biomarkers.

Further GSEA showed that, although the three hub genes were enriched in different functional pathways, they were all involved to varying degrees in aging-related processes, including mitochondrial energy metabolism, lipid metabolic remodeling, protein homeostasis, and cellular stress regulation. CKMT2 was mainly associated with the TCA cycle, adipocytokine signaling, and insulin response; ACTC1 was associated with the proteasome, RNA splicing, and DNA repair; and FOXO3 was closely related to lipid metabolic processes, including alpha-linolenic acid metabolism and ether lipid metabolism. These results suggest that FAO abnormalities in aged skeletal muscle may not represent dysregulation of a single metabolic pathway, but rather a systemic process jointly driven by impaired energy metabolism, disrupted protein quality control, and tissue structural remodeling. In addition, single-cell transcriptomic analysis further showed that the three hub genes exhibited characteristic expression patterns across different cell populations, suggesting that aging-related FAO abnormalities may not occur exclusively within muscle fibers but may also involve local microenvironmental remodeling mediated by immune and stromal cells. Emerging evidence indicates that the decline in aged skeletal muscle function is not determined solely by myocyte-intrinsic alterations but is also influenced by immune cells, pericytes, and abnormal intercellular communication [13,60]. For example, Bi et al. [60] identified a distinct SPP1^+^ macrophage subpopulation in aged skeletal muscle using single-cell RNA sequencing, suggesting that macrophage composition and inflammation-related cellular states are remodeled during aging and may contribute to alterations in the aged muscle microenvironment. In addition, Birbrair et al. [61] investigated skeletal muscle pericyte subpopulations and found that different pericyte subsets exhibit distinct differentiation potentials, with type-1 pericytes contributing to fibrous tissue deposition in aged skeletal muscle. This finding indicates that pericytes are also an important cellular source of fibrosis and tissue remodeling in aged muscle. Therefore, the scRNA-seq results of this study provide a new perspective for understanding the multicellular regulatory mechanisms underlying metabolic abnormalities in aged skeletal muscle. An increasing number of studies have shown that single algorithms are susceptible to feature collinearity and data noise. In this study, the cross-convergent strategy combining random forest, Boruta, and PPI topological analysis is consistent with current methodological trends in multi-omics feature selection and interpretable machine learning for biomarker discovery [18,62,63]. Furthermore, by integrating external dataset validation and ANN modeling, the three hub genes achieved high AUC values in both the training and validation datasets when used in combination. Therefore, from a translational perspective, this study represents a progression from candidate gene screening to combined discriminatory performance evaluation, suggesting that the three-gene signature consisting of *CKMT2*, *ACTC1*, and *FOXO3* may have greater potential than a single marker as a stratification tool for impaired FAO in aged skeletal muscle.

In addition, this study constructed an mRNA–miRNA–lncRNA regulatory network centered on *CKMT2*, *ACTC1*, and *FOXO3*, identifying 44 candidate miRNAs and 110 potential lncRNAs. These findings suggest that hub genes associated with impaired FAO in aged skeletal muscle may be jointly influenced by post-transcriptional regulatory networks. The competing endogenous RNA (ceRNA) hypothesis proposed by Salmena et al. [64] suggests that lncRNAs can regulate target mRNA expression by competitively binding miRNAs, thereby influencing cell fate and disease progression at the level of multiple genes and pathways. In skeletal muscle, lncRNA–miRNA–mRNA networks have been shown to participate widely in various physiological and pathological processes, including myogenic differentiation, muscle fiber remodeling, inflammatory regulation, and metabolic adaptation [65]. For example, hsa-miR-29a-3p identified in this study belongs to the miR-29 family, which has been closely associated with muscle fibrosis, extracellular matrix remodeling, and age-related tissue degeneration; therefore, it may be linked to ACTC1-related sarcomeric structural remodeling and cytoskeletal adaptation [66]. hsa-miR-21-3p has been associated with tissue fibrosis and inflammatory responses and can also regulate the phosphoinositide 3-kinase (PI3K)–protein kinase B (Akt) signaling pathway [67,68]. Thus, its connection with the FOXO3-related network in this study suggests that stress responses, protein degradation, and tissue remodeling in aged skeletal muscle may be coordinately regulated at the miRNA level. In addition, hsa-miR-378a-3p has been shown to activate the pyruvate–phosphoenolpyruvate (PEP) futile cycle and enhance lipolysis to improve metabolic homeostasis in mice [69]. This provides a potential upstream clue for FOXO3-mediated lipid metabolism and adaptive FAO regulation. However, it should be noted that ceRNA activity is strongly influenced by the absolute abundance of miRNAs and lncRNAs, their subcellular localization, and competitive stoichiometric relationships. Therefore, the ceRNA network identified in this study should be regarded as a predictive hypothesis requiring further validation rather than as direct mechanistic evidence [70].

DSigDB can suggest potential drug repurposing directions based on statistical associations at the gene-set level, while molecular docking and molecular dynamics simulations provide further support for the feasibility of pharmacological intervention [71,72]. In this study, candidate drugs were screened based on the DSigDB database, and molecular docking combined with 100 ns molecular dynamics simulations was further performed to evaluate the interaction characteristics between Alsterpaullone, Perhexiline, Wortmannin, and the hub proteins. The results showed that all three candidate drugs exhibited relatively low binding energies with CKMT2, ACTC1, and FOXO3. Among them, the Alsterpaullone–FOXO3, Perhexiline–FOXO3, and Alsterpaullone–CKMT2 complexes were further subjected to molecular dynamics simulation, with the Alsterpaullone–CKMT2 complex showing relatively more stable conformational characteristics. Alsterpaullone is a classical inhibitor of cyclin-dependent kinases (CDKs) and glycogen synthase kinase-3 (GSK-3), and CDK, GSK-3, and AMPK–peroxisome proliferator-activated receptor gamma coactivator 1-alpha (PGC-1α)-related networks have been implicated in the regulation of mitochondrial oxidative metabolism and muscle atrophy [73,74]. Therefore, the potential interaction of Alsterpaullone with CKMT2 or FOXO3 may provide mechanistic insights. Perhexiline is a drug related to the modulation of fatty acid oxidation, and its classical action is associated with CPT-mediated mitochondrial fatty acid entry [75], suggesting a potential metabolic connection with FAO remodeling in aged skeletal muscle. Wortmannin is an inhibitor of the PI3K pathway. Previous studies have shown that the PI3K/Akt pathway can prevent muscle atrophy by inhibiting FOXO transcription factors [76]. Therefore, Wortmannin may serve as a candidate tool molecule for dissecting the relationship between the PI3K–Akt–FOXO signaling axis and muscle atrophy. Overall, these results provide priority clues for future experimental screening of small-molecule candidates and may help advance the identified hub genes from diagnostic correlates toward mechanistic nodes and potential intervention targets.

Finally, mouse and C2C12 cellular aging models were established, and qRT-PCR validation of the hub genes was performed in gastrocnemius muscle from aged mice and D-galactose-induced C2C12 cells. The results showed expression patterns consistent with those observed in human transcriptomic datasets, further supporting the cross-species and cross-model stability of the three hub genes. Accordingly, this study established a relatively complete evidence chain from discovery in public human datasets to validation in both in vivo and in vitro experimental models, thereby enhancing the translational value of the findings.

From the perspective of human translational application, the findings of this study suggest that the three-gene combination of CKMT2, ACTC1, and FOXO3 may serve as a candidate molecular stratification tool associated with impaired FAO in aged skeletal muscle. Future translational studies should improve the specificity and reliability of this three-gene signature by using detection strategies with clearer tissue-source information. For example, the mRNA or protein expression levels of CKMT2, ACTC1, and FOXO3 could be examined in skeletal muscle biopsy samples, or their responses to aging, fatty acid metabolic stress, and exercise/nutritional interventions could be validated in human skeletal muscle cells and induced differentiated myotube models. In addition, muscle-derived extracellular vesicles, muscle-enriched transcripts or proteins, multi-omics integrated features, and multi-marker models incorporating muscle mass, grip strength, gait speed, muscle fat infiltration, cardiovascular disease status, bone mineral density, and bone turnover markers may help further distinguish skeletal muscle-derived signals and improve their clinical stratification value. Therefore, CKMT2, ACTC1, and FOXO3 should currently be interpreted as a candidate multi-gene signature associated with impaired FAO in aged skeletal muscle, rather than as single biomarkers that can be directly used for clinical diagnosis. If further confirmed in population-based studies, this signature may help identify individuals with impaired FAO-related or metabolically abnormal sarcopenia and may also serve as a potential indicator for monitoring the effects of exercise training, nutritional intervention, and metabolic modulation strategies.

This study has several limitations. First, the sample sizes of the training and validation datasets were relatively limited. Although independent validation was performed, larger population-based cohorts are still required to further confirm the stability and generalizability of these findings. Second, this study was mainly based on transcriptomic analyses, and although animal and cellular experiments validated the expression trends of the three hub genes, systematic validation at the levels of protein expression, enzyme activity, and FAO function remains lacking. Moreover, the causal roles of these genes in age-related FAO abnormalities in skeletal muscle remain to be determined. Future studies should employ gene knockout, overexpression, metabolic intervention, and enzymatic functional assays to further clarify their mechanistic roles. In addition, CKMT2, ACTC1, and FOXO3 are not strictly skeletal muscle-specific genes. CKMT2 and ACTC1 are also expressed in cardiac tissue, whereas FOXO3 is a broadly expressed stress-responsive transcription factor; therefore, their expression may be influenced by age-related comorbidities such as cardiovascular disease, myocardial injury, abnormal bone metabolism, or osteoporosis. Accordingly, these three genes should currently be regarded as a candidate multi-gene biomarker signature in the context of aged skeletal muscle rather than as stand-alone circulating diagnostic biomarkers. Future studies should combine skeletal muscle biopsy, human-derived myotube models, muscle-derived extracellular vesicles, or multi-marker models to improve tissue-source specificity and clinical interpretability. Finally, although the enrichment analyses suggested that mitochondrial metabolism, electron transport, and redox-related processes may be involved in impaired FAO in aged skeletal muscle, this study did not directly measure oxidative stress, mitochondrial density, mitochondrial respiratory function, or FAO flux. Moreover, animal validation was performed using a natural aging model under standard chow-fed conditions, and the effects of different dietary fatty acid compositions or high-fat diet conditions on these genes and FAO remodeling were not compared. Future studies should integrate oxidative stress detection, mitochondrial function assessment, FAO flux analysis, and more complete human clinical phenotyping to further validate the application value of this three-gene signature in sarcopenia, sarcopenic obesity, and intervention monitoring.

**Figure 9:**
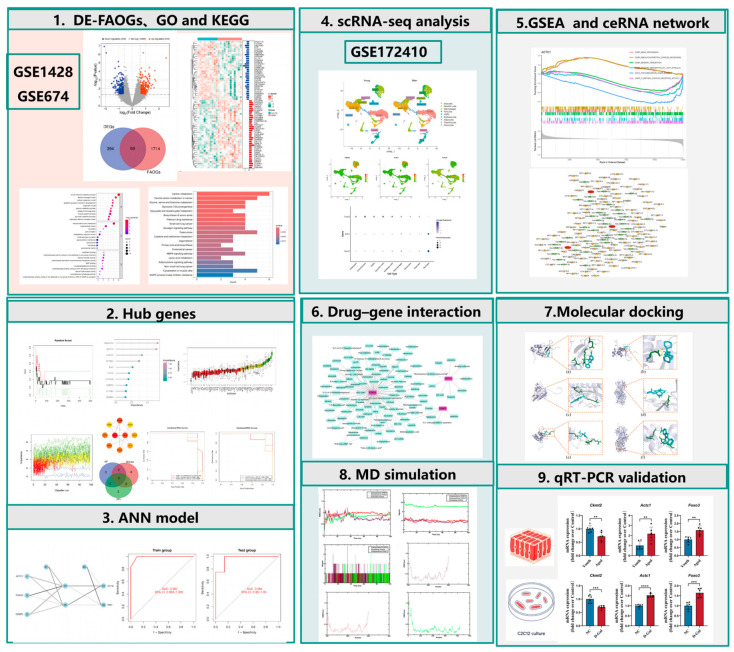
Summary of the study design, analytical workflow, and main findings. Public skeletal muscle transcriptomic datasets were used to identify differentially expressed fatty acid oxidation-related genes (DE-FAOGs), followed by functional enrichment analysis. Random forest, Boruta, and protein–protein interaction network analyses were integrated to identify the hub genes CKMT2, ACTC1, and FOXO3. An artificial neural network model was constructed to evaluate the combined predictive value of the three genes. Single-cell RNA sequencing analysis, GSEA, ceRNA network construction, drug–gene interaction prediction, molecular docking, molecular dynamics simulation, and qRT-PCR validation were further performed to explore their biological relevance, regulatory features, potential drug interactions, and expression consistency in aging models. Data are presented as the mean ± SD. ** *p* < 0.01, *** *p* < 0.001, **** *p* < 0.0001.

Given the large number of analytical modules and results included in this study, we generated a summary figure to provide an integrated overview of the study design, analytical workflow, and main findings (Figure 9). This figure summarizes the identification of DE-FAOGs, hub gene screening, ANN model construction, single-cell expression analysis, GSEA and ceRNA network analysis, drug–gene interaction prediction, molecular docking, molecular dynamics simulation, and qRT-PCR validation, thereby helping readers better understand the logical relationship among the multi-layered analyses performed in this study.

## 5. Conclusions

This study used impaired FAO as the entry point and systematically identified key biomarkers associated with skeletal muscle aging by integrating bioinformatics analysis, machine learning algorithms, single-cell transcriptomics, and experimental validation. The results demonstrated that CKMT2, ACTC1, and FOXO3 are important hub genes associated with fatty acid oxidation abnormalities in aged skeletal muscle, involving key biological processes such as mitochondrial energy metabolism, muscle fiber structural remodeling, and stress- and metabolism-related regulation. The ANN model constructed based on these three genes showed good discriminatory performance in both the training and validation datasets, suggesting their potential diagnostic value. Furthermore, the expression patterns of these genes were validated in naturally aged mouse skeletal muscle tissues and a D-galactose-induced C2C12 cellular senescence model. Collectively, this study reveals molecular features associated with fatty acid oxidation dysfunction in aged skeletal muscle and supports the potential value of CKMT2, ACTC1, and FOXO3 as biomarkers. These findings provide a theoretical basis for further elucidating the metabolic mechanisms of skeletal muscle aging and sarcopenia and for developing new strategies for early identification and intervention.

## Figures and Tables

**Table 1:** Details of the datasets used in this study.

Data Set	Samples	Platform	Tissue	Group
GSE1428	12 older vs. 10 young	GPL96	Vastus lateralis muscle	Training set
GSE674	8 older vs. 7 young	GPL96	Vastus lateralis muscle	Test set

**Table 2:** List of primers used for qRT-PCR.

Gene	Forward Primer Sequences	Reverse Primer Sequences
Ckmt2	5′-CAAACTCCGAAACAAGATG-3′	5′-GTACCCGAGAGGACAACAC-3′
Actc1	5′-TGGATTTTGAGAACGAGA-3′	5′-CATACCAATGAAAGAGGG-3′
Foxo3	5′-GTCGTCTTGTGTTTGTTTCCTT-3′	5′-TGTGCCATTGTTCAGTTTTTAG-3′
Hprt	5′-CTCATGGACTGATTATGGACAGGAC-3′	5′-GCAGGTCAGCAAAGAACTTATAGCC-3′

**Table 3:** Binding energy between key drugs and hub genes (kcal/mol).

Key Drugs	Hub Genes		
	ACTC1	CKMT2	FOXO3
Alsterpaullone	−6.65	−7.12	−7.57
Perhexiline	−5.56	−5.37	−7.24
Wortmannin	−6.14	−5.62	−7.07

## Data Availability

The datasets analyzed in this study are publicly available in the GEO database under accession numbers GSE1428, GSE674, and GSE172410. Additional data supporting the findings of this study are provided in the Supplementary Materials.

## Supplementary Materials

The following supporting information can be downloaded at: https://www.mdpi.com/article/10.3390/biom16071030/s1, Supplementary Material S1: Table S1: 69 Differentially expressed FAOGs; Table S2a: The top 10 items of BP, CC, and MF; Table S2b: The top 20 items of KEGG; Table S3a: RF genes; Table S3b: Boruta genes; Table S3c: PPI genes; Table S3d: InterGenes; Table S4a: Gene-miRNA; Table S4b: miRNA-lncRNA; Table S5: Drugs prediction. Supplementary Material S2: List of fatty acid oxidation-related protein-coding genes (FAOGs).
