# Supplementary material for: Identification of Potential Biomarkers Associated with Impaired Fatty Acid Oxidation in Aged Skeletal Muscle Using Bioinformatics and Machine Learning Approaches
Source: Biomolecules. 2026 Jul 14;16(7):1030. doi: 10.3390/biom16071030 (PMC13406276; doi:10.3390/biom16071030)
Supplement: Supplementary file 1 [file biomolecules-16-01030-s001.zip › Supplementary Material S1.pdf]

Supplementary Material S1

Contents of supplementary material S1

Table S1. 69 Differentially expressed FAOGs. 1
Table S2a. The top 10 items of BP, CC, and MF. 2
Table S2b. The top 20 items of KEGG. 3
Table S3a.RF genes. 5
Table S3b. Boruta genes. 5
Table S3c. PPI genes. 5
Table S3d. InterGenes. 5
Table S4a. Gene-miRNA. 5
Table S4b. miRNA- lncRNA. 9
Table S5. Drugs prediction. 12

Table S1. 69 Differentially expressed FAOGs.

PFKP	DECR1	CYCS	NKX2-5	COL4A5	NDUFAF4
DNAJC15	COQ9	DNMT3B	ACTC1	KDM5A	MFAP5

MANF	MYOZ2	LGR5	PHGDH	PKP2	FKTN
SLC31A1	SLC25A4	ERBB2	MBTPS2	GLDC	PPRC1
NFKBIA	UCP3	CKMT2	SLIT2	PGAM2	MOCS2
ACOT8	KCNJ8	SI	SOAT2	MALT1	HAO1
GCM2	PUS1	HTR3B	RAB23	LDHA	TCTN1
CIDEA	FBN1	CHKA	ADA	ITGAM	ETFDH
FLT3	UCP1	CYP27A1	CCKAR	TNFRSF1B	ASNS
MC5R	CYP2C8	SLC35A3	DLD	PEX11A	RPGR
SLC5A2	ACD	FANCE	FOXO3	CEBPB	BAX
CP	ATP8B1	PRKAG1			

Table S2a. The top 10 items of BP, CC, and MF.

Description	enrichment(-log10(p))	pvalue	Count	ONTOLOGY
small molecule catabolic process	6.015511959	9.64913E-07	10	BP
electron transport chain	5.461277507	3.45718E-06	7	BP
cellular response to cold	4.886081616	1.29993E-05	3	BP
apoptotic process involved in development	4.869787796	1.34962E-05	4	BP
response to cold	4.78412871	1.64388E-05	4	BP
glycine metabolic process	4.514491066	3.0585E-05	3	BP
adaptive thermogenesis	4.452165721	3.53048E-05	6	BP
muscle system process	4.371331745	4.25273E-05	9	BP
pyruvate metabolic process	4.146372649	7.13884E-05	5	BP
respiratory electron transport chain	4.06083681	8.69287E-05	5	BP
mitochondrial inner membrane	5.089812163	8.13E-06	10	CC
mitochondrial matrix	3.536921675	0.000290455	8	CC

microfibril	3.04469582	0.000902203	2	CC
pore complex	2.437545847	0.003651356	2	CC
endoplasmic reticulum lumen	2.335888012	0.004614365	5	CC
oxidoreductase complex	1.972369409	0.010656893	3	CC
apical plasma membrane	1.891549465	0.012836616	5	CC
peroxisome	1.870082495	0.013487067	3	CC
microbody	1.870082495	0.013487067	3	CC
peroxisomal matrix	1.868109093	0.01354849	2	CC
cardiolipin binding	3.145693571	0.000715001	2	MF
oxidoreductase activity, acting on the aldehyde or oxo group of donors	3.138281136	0.000727309	3	MF
electron transfer activity	3.044845862	0.000901891	4	MF
carbohydrate derivative transmembrane transporter activity	2.942531129	0.001141481	3	MF
AMP binding	2.868987862	0.00135211	2	MF
phosphatidylglycerol binding	2.868987862	0.00135211	2	MF
oxidoreductase activity, acting on CH-OH group of donors	2.814671441	0.001532246	4	MF
steroid hydroxylase activity	2.389604089	0.004077518	2	MF
intramolecular transferase activity	2.326048555	0.004720103	2	MF
oxidoreductase activity, acting on the aldehyde or oxo group of donors, NAD or NADP as acceptor	2.0449937	0.009015842	2	MF

Table S2b. The top 20 items of KEGG.

ID	Description	GeneRatio	BgRatio	pvalue	qvalue	Count
hsa01200	Carbon metabolism	6/51	116/9376	3.60304E-05	0.003116125	6

hsa05230	Central carbon metabolism in cancer	5/51	71/9376	3.8697E-05	0.003116125	5
hsa00260	Glycine, serine and threonine metabolism	4/51	41/9376	6.77897E-05	0.003639234	4
hsa00010	Glycolysis / Gluconeogenesis	4/51	67/9376	0.00046236	0.016338016	4
hsa00630	Glyoxylate and dicarboxylate metabolism	3/51	31/9376	0.000612163	0.016338016	3
hsa01230	Biosynthesis of amino acids	4/51	75/9376	0.000710116	0.016338016	4
hsa01524	Platinum drug resistance	4/51	75/9376	0.000710116	0.016338016	4
hsa05222	Small cell lung cancer	4/51	93/9376	0.001587555	0.031959988	4
hsa04922	Glucagon signaling pathway	4/51	107/9376	0.002653704	0.045296323	4
hsa05152	Tuberculosis	5/51	181/9376	0.002904662	0.045296323	5
hsa00270	Cysteine and methionine metabolism	3/51	54/9376	0.003093768	0.045296323	3
hsa05134	Legionellosis	3/51	56/9376	0.003431214	0.045683951	3
hsa00120	Primary bile acid biosynthesis	2/51	17/9376	0.003744608	0.045683951	2
hsa05213	Endometrial cancer	3/51	59/9376	0.003978261	0.045683951	3
hsa04152	AMPK signaling pathway	4/51	122/9376	0.004254878	0.045683951	4
hsa00785	Lipoic acid metabolism	2/51	19/9376	0.004675718	0.047064795	2
hsa04920	Adipocytokine signaling pathway	3/51	70/9376	0.006424132	0.060860194	3
hsa05223	Non-small cell lung cancer	3/51	73/9376	0.007216401	0.064567796	3
hsa04820	Cytoskeleton in muscle cells	5/51	233/9376	0.008416101	0.071338699	5
hsa01521	EGFR tyrosine kinase inhibitor resistance	3/51	80/9376	0.00928275	0.073726619	3

Table S3a.RF genes.

DNAJC15	ACTC1	FOXO3	ETFDH	DLD
CYCS	CYP2C8	CKMT2	HTR3B	KDM5A

Table S3b. Boruta genes.

ACTC1	DNAJC15	CEBPB	SLIT2	NFKBIA	FOXO3
CYP27A1	CKMT2	ETFDH	PGAM2	KCNJ8	

Table S3c. PPI genes.

ACTC1	FOXO3	CYCS	NFKBIA	CEBPB
PHGDH	LDHA	ERBB2	CKMT2	DLD

Table S3d. InterGenes.

CKMT2	ACTC1	FOXO3

Table S4a. Gene-miRNA.

Gene	miRNA	miRanda	miRDB	TargetScan	Sum
CKMT2	hsa-miR-634	1	1	1	3
CKMT2	hsa-miR-922	1	1	1	3
CKMT2	hsa-miR-1324	1	1	1	3
CKMT2	hsa-miR-3173-3p	1	1	1	3
CKMT2	hsa-miR-590-3p	1	1	1	3
CKMT2	hsa-miR-654-3p	1	1	1	3
CKMT2	hsa-miR-940	1	1	1	3
CKMT2	hsa-miR-4330	1	1	1	3
CKMT2	hsa-miR-3166	1	1	1	3
ACTC1	hsa-let-7g-3p	1	1	1	3
ACTC1	hsa-miR-4272	1	1	1	3
ACTC1	hsa-miR-551b-5p	1	1	1	3

ACTC1	hsa-miR-92b-3p	1	1	1	3
ACTC1	hsa-miR-513c-5p	1	1	1	3
ACTC1	hsa-miR-548b-3p	1	1	1	3
ACTC1	hsa-miR-223-5p	1	1	1	3
ACTC1	hsa-miR-570-3p	1	1	1	3
ACTC1	hsa-miR-4325	1	1	1	3
ACTC1	hsa-miR-495-3p	1	1	1	3
ACTC1	hsa-miR-4297	1	1	1	3
ACTC1	hsa-miR-30b-5p	1	1	1	3
ACTC1	hsa-miR-892a	1	1	1	3
ACTC1	hsa-miR-7-5p	1	1	1	3
ACTC1	hsa-miR-3171	1	1	1	3
ACTC1	hsa-miR-561-3p	1	1	1	3
ACTC1	hsa-miR-32-5p	1	1	1	3
ACTC1	hsa-miR-576-5p	1	1	1	3
ACTC1	hsa-miR-25-3p	1	1	1	3
ACTC1	hsa-miR-548g-3p	1	1	1	3
ACTC1	hsa-miR-139-5p	1	1	1	3
ACTC1	hsa-miR-557	1	1	1	3
ACTC1	hsa-miR-548x-3p	1	1	1	3
ACTC1	hsa-miR-299-5p	1	1	1	3
ACTC1	hsa-miR-221-3p	1	1	1	3
ACTC1	hsa-miR-3119	1	1	1	3
ACTC1	hsa-let-7a-2-3p	1	1	1	3
ACTC1	hsa-miR-3182	1	1	1	3
ACTC1	hsa-miR-568	1	1	1	3
ACTC1	hsa-miR-29b-1-5p	1	1	1	3
ACTC1	hsa-miR-373-5p	1	1	1	3
ACTC1	hsa-miR-222-3p	1	1	1	3
ACTC1	hsa-miR-507	1	1	1	3
ACTC1	hsa-miR-30c-5p	1	1	1	3
ACTC1	hsa-miR-514b-5p	1	1	1	3
ACTC1	hsa-miR-938	1	1	1	3
ACTC1	hsa-miR-4264	1	1	1	3
ACTC1	hsa-miR-429	1	1	1	3
ACTC1	hsa-miR-340-5p	1	1	1	3
ACTC1	hsa-miR-363-3p	1	1	1	3
ACTC1	hsa-miR-367-3p	1	1	1	3
ACTC1	hsa-miR-142-5p	1	1	1	3
ACTC1	hsa-miR-30d-5p	1	1	1	3
FOXO3	hsa-miR-518a-5p	1	1	1	3
FOXO3	hsa-miR-644a	1	1	1	3
FOXO3	hsa-miR-3168	1	1	1	3
FOXO3	hsa-miR-132-3p	1	1	1	3

FOXO3	hsa-miR-212-3p	1	1	1	3
FOXO3	hsa-miR-1202	1	1	1	3
FOXO3	hsa-miR-302e	1	1	1	3
FOXO3	hsa-miR-181c-3p	1	1	1	3
FOXO3	hsa-let-7b-3p	1	1	1	3
FOXO3	hsa-miR-384	1	1	1	3
FOXO3	hsa-miR-4255	1	1	1	3
FOXO3	hsa-miR-3143	1	1	1	3
FOXO3	hsa-miR-29b-3p	1	1	1	3
FOXO3	hsa-miR-1299	1	1	1	3
FOXO3	hsa-miR-524-5p	1	1	1	3
FOXO3	hsa-miR-491-3p	1	1	1	3
FOXO3	hsa-miR-1247-5p	1	1	1	3
FOXO3	hsa-miR-302a-3p	1	1	1	3
FOXO3	hsa-miR-891b	1	1	1	3
FOXO3	hsa-miR-507	1	1	1	3
FOXO3	hsa-miR-4279	1	1	1	3
FOXO3	hsa-miR-182-5p	1	1	1	3
FOXO3	hsa-miR-1915-3p	1	1	1	3
FOXO3	hsa-miR-302b-3p	1	1	1	3
FOXO3	hsa-miR-9-5p	1	1	1	3
FOXO3	hsa-miR-584-5p	1	1	1	3
FOXO3	hsa-miR-93-3p	1	1	1	3
FOXO3	hsa-miR-373-3p	1	1	1	3
FOXO3	hsa-miR-27a-3p	1	1	1	3
FOXO3	hsa-miR-520c-3p	1	1	1	3
FOXO3	hsa-miR-4328	1	1	1	3
FOXO3	hsa-miR-513a-5p	1	1	1	3
FOXO3	hsa-miR-590-3p	1	1	1	3
FOXO3	hsa-miR-520a-3p	1	1	1	3
FOXO3	hsa-let-7f-2-3p	1	1	1	3
FOXO3	hsa-miR-3065-3p	1	1	1	3
FOXO3	hsa-miR-361-3p	1	1	1	3
FOXO3	hsa-miR-767-5p	1	1	1	3
FOXO3	hsa-miR-96-5p	1	1	1	3
FOXO3	hsa-miR-138-2-3p	1	1	1	3
FOXO3	hsa-let-7f-1-3p	1	1	1	3
FOXO3	hsa-miR-708-3p	1	1	1	3
FOXO3	hsa-miR-527	1	1	1	3
FOXO3	hsa-miR-1909-3p	1	1	1	3
FOXO3	hsa-miR-588	1	1	1	3
FOXO3	hsa-miR-302d-3p	1	1	1	3
FOXO3	hsa-let-7a-3p	1	1	1	3
FOXO3	hsa-miR-557	1	1	1	3

FOXO3	hsa-miR-362-3p	1	1	1	3
FOXO3	hsa-miR-1283	1	1	1	3
FOXO3	hsa-miR-4271	1	1	1	3
FOXO3	hsa-miR-551b-5p	1	1	1	3
FOXO3	hsa-miR-574-5p	1	1	1	3
FOXO3	hsa-miR-300	1	1	1	3
FOXO3	hsa-miR-196a-3p	1	1	1	3
FOXO3	hsa-miR-10b-3p	1	1	1	3
FOXO3	hsa-miR-539-5p	1	1	1	3
FOXO3	hsa-miR-593-3p	1	1	1	3
FOXO3	hsa-miR-545-5p	1	1	1	3
FOXO3	hsa-miR-302c-5p	1	1	1	3
FOXO3	hsa-miR-3120-3p	1	1	1	3
FOXO3	hsa-miR-1293	1	1	1	3
FOXO3	hsa-miR-27b-3p	1	1	1	3
FOXO3	hsa-miR-495-3p	1	1	1	3
FOXO3	hsa-miR-1271-5p	1	1	1	3
FOXO3	hsa-miR-590-5p	1	1	1	3
FOXO3	hsa-miR-518d-5p	1	1	1	3
FOXO3	hsa-miR-3125	1	1	1	3
FOXO3	hsa-miR-4284	1	1	1	3
FOXO3	hsa-miR-4272	1	1	1	3
FOXO3	hsa-miR-146a-3p	1	1	1	3
FOXO3	hsa-miR-21-5p	1	1	1	3
FOXO3	hsa-miR-1303	1	1	1	3
FOXO3	hsa-miR-1258	1	1	1	3
FOXO3	hsa-miR-548a-3p	1	1	1	3
FOXO3	hsa-miR-3065-5p	1	1	1	3
FOXO3	hsa-miR-29c-3p	1	1	1	3
FOXO3	hsa-miR-29a-3p	1	1	1	3
FOXO3	hsa-miR-381-3p	1	1	1	3
FOXO3	hsa-miR-148b-5p	1	1	1	3
FOXO3	hsa-miR-26b-3p	1	1	1	3
FOXO3	hsa-miR-577	1	1	1	3
FOXO3	hsa-miR-1208	1	1	1	3
FOXO3	hsa-miR-223-3p	1	1	1	3
FOXO3	hsa-miR-340-5p	1	1	1	3
FOXO3	hsa-miR-548g-3p	1	1	1	3
FOXO3	hsa-miR-1183	1	1	1	3
FOXO3	hsa-miR-21-3p	1	1	1	3
FOXO3	hsa-miR-1305	1	1	1	3
FOXO3	hsa-miR-2278	1	1	1	3
FOXO3	hsa-miR-28-3p	1	1	1	3
FOXO3	hsa-miR-3123	1	1	1	3

FOXO3	hsa-miR-186-3p	1	1	1	3
FOXO3	hsa-miR-520d-3p	1	1	1	3
FOXO3	hsa-miR-421	1	1	1	3
FOXO3	hsa-miR-130a-5p	1	1	1	3
FOXO3	hsa-miR-466	1	1	1	3
FOXO3	hsa-miR-450b-5p	1	1	1	3
FOXO3	hsa-miR-448	1	1	1	3

Table S4b. miRNA- lncRNA.

miRNA	lncRNA
hsa-miR-7-5p	CDR1-AS
hsa-miR-1208	FLJ16779
hsa-miR-767-5p	RP11-326C3.10
hsa-miR-570-3p	RP11-10J21.4
hsa-miR-1202	HP09025
hsa-miR-7-5p	RP11-830F9.6
hsa-miR-27a-3p	RP11-10J21.4
hsa-miR-421	AC079779.7
hsa-miR-223-5p	AC069257.8
hsa-miR-1324	AC079779.7
hsa-miR-26b-3p	LA16c-OS12.2
hsa-miR-767-5p	RP11-326C3.14
hsa-miR-181c-3p	CTD-2534I21.9
hsa-miR-7-5p	FLJ35934
hsa-miR-146a-3p	FAM74A1
hsa-miR-146a-3p	RP11-830F9.6
hsa-let-7a-3p	RP3-323A16.1
hsa-miR-223-5p	RP3-323A16.1
hsa-miR-146a-3p	FAM74A7
hsa-miR-146a-3p	FAM74A6
hsa-let-7a-3p	DPP10-AS2
hsa-miR-361-3p	RP3-470B24.5
hsa-miR-576-5p	CTC-459F4.1
hsa-miR-892a	GAS6-AS1
hsa-miR-93-3p	RP5-892K4.1
hsa-miR-7-5p	RP11-338K13.1
hsa-miR-130a-5p	LL22NC03-27C5.1
hsa-miR-7-5p	RP11-932O9.4
hsa-miR-1324	RP4-737E23.2
hsa-miR-361-3p	RP13-507P19.2
hsa-miR-450b-5p	CTC-265F19.1

hsa-let-7a-3p	FAM230B
hsa-miR-181c-3p	RP11-627G23.1
hsa-miR-302a-3p	RP4-539M6.22
hsa-miR-1208	RP11-431K24.1
hsa-miR-223-3p	FAM95B1
hsa-miR-421	LINC01165
hsa-miR-7-5p	AC006019.3
hsa-miR-561-3p	RP11-231G3.1
hsa-miR-593-3p	CTD-2532K18.2
hsa-miR-223-5p	LINC00689
hsa-miR-1208	C22orf34
hsa-miR-223-5p	RP11-426C22.4
hsa-miR-223-5p	HPVC1
hsa-miR-27a-3p	RP11-449D8.5
hsa-miR-539-5p	AC018816.3
hsa-miR-593-3p	AC005330.2
hsa-let-7a-2-3p	AC011718.2
hsa-miR-223-5p	RP11-243A14.1
hsa-miR-93-3p	RP11-130L8.1
hsa-miR-27a-3p	LINC01123
hsa-miR-130a-5p	RP11-210M15.1
hsa-miR-139-5p	AC015849.16
hsa-miR-26b-3p	RP13-143G15.4
hsa-miR-590-3p	LINC00240
hsa-miR-130a-5p	AC084219.4
hsa-miR-593-3p	CTD-2553C6.1
hsa-miR-593-3p	MZF1-AS1
hsa-miR-182-5p	RP11-34P13.7
hsa-miR-146a-3p	LINC00689
hsa-miR-1324	LINC01070
hsa-miR-7-5p	RP11-394A14.2
hsa-miR-590-3p	AC005614.3
hsa-miR-181c-3p	SNHG14
hsa-miR-223-3p	RP1-182D15.2
hsa-miR-539-5p	ZNF883
hsa-miR-539-5p	CTC-435M10.10
hsa-miR-93-3p	CAMTA1-IT1
hsa-miR-139-5p	RP11-231D20.2
hsa-miR-182-5p	FENDRR
hsa-miR-29a-3p	RP11-223P11.3
hsa-miR-767-5p	RP11-223P11.3
hsa-miR-361-3p	HOXC-AS1
hsa-miR-182-5p	AC010524.2
hsa-miR-181c-3p	CTD-2281E23.3

hsa-miR-7-5p	LINC00662
hsa-miR-590-3p	RP11-762H8.4
hsa-miR-767-5p	MCF2L-AS1
hsa-miR-27a-3p	CTD-2281E23.1
hsa-miR-21-3p	RP11-130C6.1
hsa-miR-130a-5p	AC068489.1
hsa-miR-361-3p	RP11-158I9.8
hsa-miR-593-3p	AC137934.1
hsa-miR-590-3p	AC006548.28
hsa-miR-93-3p	CTB-50L17.7
hsa-miR-590-3p	AC093639.1
hsa-miR-93-3p	RP11-673P17.2
hsa-miR-518d-5p	CTC-273B12.5
hsa-miR-340-5p	LINC00869
hsa-miR-539-5p	SATB1-AS1
hsa-miR-93-3p	LINC01529
hsa-miR-130a-5p	LINC00664
hsa-miR-539-5p	LINC01539
hsa-miR-892a	C22orf34
hsa-miR-299-5p	AC011284.3
hsa-miR-361-3p	RP11-44M6.7
hsa-miR-361-3p	RP11-561O23.5
hsa-miR-9-5p	RP11-397O4.1
hsa-let-7a-3p	LPP-AS2
hsa-miR-539-5p	RP11-598F7.3
hsa-miR-938	RP11-989E6.10
hsa-miR-590-3p	CTD-2561J22.5
hsa-miR-361-3p	RP4-751H13.7
hsa-miR-518a-5p	CTD-2521M24.5
hsa-miR-708-3p	LINC00662
hsa-miR-28-3p	LINC00662
hsa-miR-1202	SNHG14
hsa-miR-7-5p	CTA-243E7.1
hsa-miR-518d-5p	CTC-548K16.6
hsa-miR-654-3p	FAM230B
hsa-miR-590-3p	LA16c-60D12.2
hsa-miR-767-5p	RP4-539M6.22
hsa-miR-340-5p	RP11-374A4.1
hsa-miR-27a-3p	AC078942.1
hsa-miR-539-5p	EGFLAM-AS3
hsa-miR-186-3p	RP11-368I7.4
hsa-miR-590-3p	RP11-638L3.1
hsa-miR-922	LA16c-306A4.2
hsa-miR-574-5p	RP5-894D12.5

hsa-miR-940	RP11-458F8.4
hsa-let-7f-2-3p	FAM230B
hsa-miR-922	RP4-539M6.22
hsa-miR-940	AP001476.4
hsa-miR-940	LINC00265

Table S5. Drugs prediction.

Term	Genes	type
Monoisoamyl-2,3-dimercaptosuccinate	ACTC1	drug_prediction
estriol	ACTC1	drug_prediction
alsterpaullone	ACTC1	drug_prediction
folic acid	ACTC1	drug_prediction
dimethyl sulfoxide	ACTC1	drug_prediction
carmustine	ACTC1	drug_prediction
Dasatinib	ACTC1	drug_prediction
cytarabine	ACTC1	drug_prediction
Caspan	ACTC1	drug_prediction
3-(1-methylpyrrolidin-2-yl)pyridine	CKMT2	drug_prediction
8-Bromo-cAMP, Na	CKMT2	drug_prediction
Fonofos	CKMT2	drug_prediction
TERBUFOS	CKMT2	drug_prediction
parathion	CKMT2	drug_prediction
2,2',4,5,5'-PENTACHLOROBIPHENYL	FOXO3	drug_prediction
resveratrol	FOXO3	drug_prediction
Resveratrol-13C6	FOXO3	drug_prediction
Lapatinib	FOXO3	drug_prediction
nadide	FOXO3	drug_prediction
H-89	FOXO3	drug_prediction
170449-18-0	FOXO3	drug_prediction
wortmannin	FOXO3	drug_prediction
nicotinamide	FOXO3	drug_prediction
Octa-2,4,6-trienoic acid	FOXO3	drug_prediction
pimozide	FOXO3	drug_prediction
7,8-Benzoflavone	FOXO3	drug_prediction
Gefitinib	FOXO3	drug_prediction
picrotoxinin	FOXO3	drug_prediction
Nandrolone phenpropionate	FOXO3	drug_prediction
perhexiline	FOXO3	drug_prediction
niclosamide	FOXO3	drug_prediction
AG-012559	FOXO3	drug_prediction
mefloquine	FOXO3	drug_prediction

prenylamine	FOXO3	drug_prediction
Zinc acetate dihydrate	FOXO3	drug_prediction
astemizole	FOXO3	drug_prediction
dUTP	FOXO3	drug_prediction
doxycycline	FOXO3	drug_prediction
L-threonine	FOXO3	drug_prediction
calmidazolium	FOXO3	drug_prediction
PROPIDIUM	FOXO3	drug_prediction
L-leucine	FOXO3	drug_prediction
terfenadine	FOXO3	drug_prediction
Ethylparaben	FOXO3	drug_prediction
Imatinib mesylate	FOXO3	drug_prediction
hydrogen peroxide	FOXO3	drug_prediction
metformin	FOXO3	drug_prediction
streptozocin	FOXO3	drug_prediction
N-Acetyl-L-cysteine	FOXO3	drug_prediction
Insulin	FOXO3	drug_prediction
1-Phosphatidyl-myo-inositol	FOXO3	drug_prediction
17-Hydroxy-17-methylandrost-4-en-3-one	FOXO3	drug_prediction
PLATINUM	FOXO3	drug_prediction
2-Butanone	FOXO3	drug_prediction
fulvestrant	FOXO3	drug_prediction
rapamycin	FOXO3	drug_prediction
doxorubicin	FOXO3	drug_prediction
dexamethasone	FOXO3	drug_prediction
2,2',4,4',5,5'-Hexachlorobiphenyl	FOXO3	drug_prediction
LY 294002	FOXO3	drug_prediction
nitrofural	FOXO3	drug_prediction
ethanol	FOXO3	drug_prediction
3,3',4,4',5-Pentachlorobiphenyl	FOXO3	drug_prediction
dorzolamide	FOXO3	drug_prediction
puromycin	FOXO3	drug_prediction
TPEN	FOXO3	drug_prediction
PNU-0293363	FOXO3	drug_prediction
curcumin	FOXO3	drug_prediction
PD 98059	FOXO3	drug_prediction
MG-132	FOXO3	drug_prediction
PHA-00665752	FOXO3	drug_prediction
Zinc sulfate	FOXO3	drug_prediction
digoxigenin	FOXO3	drug_prediction
podophyllotoxin	FOXO3	drug_prediction
helveticoside	FOXO3	drug_prediction
mifepristone	FOXO3	drug_prediction
cicloheximide	FOXO3	drug_prediction

Medroxyprogesterone acetate	FOXO3	drug_prediction
NICKEL CHLORIDE	FOXO3	drug_prediction
Methaneseleninic acid	FOXO3	drug_prediction
lanatoside C	FOXO3	drug_prediction
Pentabromodiphenyl ether	FOXO3	drug_prediction
ouabain	FOXO3	drug_prediction
diclofenac	FOXO3	drug_prediction
deptropine	FOXO3	drug_prediction
carbamazepine	FOXO3	drug_prediction
strophanthidin	FOXO3	drug_prediction
etoposide	FOXO3	drug_prediction
azacitidine	FOXO3	drug_prediction
Cylindrospermopsin	FOXO3	drug_prediction
Phorbol 12-myristate 13-acetate	FOXO3	drug_prediction
digoxin	FOXO3	drug_prediction
GDC-0941	FOXO3	drug_prediction
chlorhexidine	FOXO3	drug_prediction
etifenin	FOXO3	drug_prediction
staurosporine	FOXO3	drug_prediction
neostigmine bromide	FOXO3	drug_prediction
8-Bromo-cAMP, Na	FOXO3	drug_prediction
meclofenoxate	FOXO3	drug_prediction
anisomycin	FOXO3	drug_prediction
metronidazole	FOXO3	drug_prediction
ampyrone	FOXO3	drug_prediction
emetine	FOXO3	drug_prediction
captopril	FOXO3	drug_prediction
lycorine	FOXO3	drug_prediction
Cianidanol	FOXO3	drug_prediction
cimetidine	FOXO3	drug_prediction
Enterolactone	FOXO3	drug_prediction
